# A prolonged hydrothermal past at Santorini Caldera revealed by sedimentary trace metal and microbial signatures

**DOI:** 10.1038/s41467-026-75931-8

**Published:** 2026-08-04

**Authors:** Sofia Della Sala, Vasiliki Papadimitriou, Paraskevi Polymenakou, Steffen Kutterolf, Joost Frieling, David M. Pyle, Tamsin A. Mather, Stephanos Kilias, Christopher K. Jones, Tim Druitt, Jonas Preine, Christian Hübscher, Paraskevi Nomikou, Olga Koukousioura, Thomas A. Ronge, Sarah Beethe, Katharina Pank, Carole Berthod, Hehe Chen, Shun Chiyonobu, Acacia Clark, Susan DeBari, Ralf Gertisser, Raymond Johnston, Michael Manga, Molly McCanta, Iona McIntosh, Ally Peccia, Masako Tominaga, Yuzuru Yamamoto, Adam Woodhouse, Alexis Bernard, Tatiana Fernandez Perez, Kumar Batuk Joshi, Günther Kletetschka, Antony Morris, Xiaohui Li, Dimitrios Papanikolaou

**Affiliations:** 1 Department of Earth Sciences, University of Oxford, Oxford, United Kingdom; 2 Hellenic Centre for Marine Research, Institute of Marine Biology, Biotechnology and Aquaculture, Crete, Greece; 3 Department of Geology & Geoenvironment, National & Kapodistrian, University of Athens, Athens, Greece; 4 GEOMAR Helmholtz Centre for Ocean Research, Kiel, Germany; 5 Department of Geology, Ghent University, Ghent, Belgium; 6 Department of Earth and Planetary Sciences, University of California, Riverside, CA USA; 7 University Clermont-Auvergne, CNRS, IRD, OPGC, Laboratoire Magmas et Volcans, Clermont-Ferrand, France; 8 National Oceanographic Centre, Southampton, United Kingdom; 9 Department of Geology and Geophysics, Woods Hole Oceanographic Institution, Woods Hole, MA USA; 10 Institute of Geophysics, University of Hamburg, Hamburg, Germany; 11 School of Geology, Aristotle University of Thessaloniki, Thessaloniki, Greece; 12 Texas A&M University, College Station, TX USA; 13 College of Earth, Ocean, and Atmospheric Sciences, Oregon State University, Corvallis, OR USA; 14 Institut De Physique Du Globe De Paris, Centre National de la Recherche Scientifique (CNRS), Paris, France; 15 School of Ocean Sciences, China University of Geosciences, Beijing, China; 16 Faculty of International Resource Sciences, Akita University, Akita, Japan; 17 School of Natural Sciences/CODES, University of Tasmania, Hobart, TAS Australia; 18 Geology Department, Western Washington University, Bellingham, WA USA; 19 School of Life Sciences, Keele University, Keele, UK; 20 School of Geosciences, University of South Florida, Tampa, FL USA; 21 Department of Earth and Planetary Science, University of California, Berkeley, CA USA; 22 Department of Earth and Planetary Sciences, University of Tennessee, Knoxville, TN USA; 23 Japan Agency for Marine-Earth Science and Technology, Yokosuka, Japan; 24 Lamont-Doherty Earth Observatory, Columbia University, Palisades, NY USA; 25 Graduate School of Science, Kobe University, Kobe, Japan; 26 School of Earth and Environmental Sciences, Cardiff University, Cardiff, UK; 27 Laboratoire des Fluides Complexes et leurs Réservoirs, Université de Pau et des Pays de l’Adour, Pau, France; 28 Department of Geology, Kent State University, Kent, OH USA; 29 Department of Geology, Central University of Himachal Pradesh, Kangra, India; 30 Institute of Hydrogeology, Engineering Geology and Applied Geophysics, Faculty of Science, Charles University, Prague, Czechia; 31 Geophysical Institute, University of Alaska, Fairbanks, AK USA; 32 School of Geography, Earth and Environmental Sciences, Plymouth University, Plymouth, UK; 33 Key Laboratory of Submarine Geoscience and Prospecting Techniques, Ocean University of China, Qingdao, China

**Keywords:** Element cycles, Volcanology, Sedimentology, Geochemistry, Geochemistry

## Abstract

Hydrothermal systems in volcanic calderas are critical in signalling volcanic unrest, forming ore deposits, and sustaining chemosynthetic microorganisms. Analysis of a ~3500-year sequence of sediments collected from the Santorini caldera, Greece, during International Ocean Discovery Program (IODP) Expedition 398 reveals the behaviour of a prolonged paleo-hydrothermal system. Sediment geochemical and metagenomic data record vigorous hydrothermal activity and metal fluxes for ~1100 years, within a 2270-year window between two major eruptions. Sediment hydrothermally-derived trace metals are significantly enriched over background (~200-fold for As and Hg, and 10–50-fold for Mn, Sb, Mo, and V), with long-term metal fluxes (9 t yr^−1^ As, 2.5 t yr^−1^ Cu, 7 kg yr^−1^ Ag) comparable to fluxes from present-day geothermal fields in the Taupo Volcanic Zone. Metagenomic analysis identifies elevated metal resistance genes—signals of microbial adaptation to heightened hydrothermal stressors. Here we integrate geological and genomic evidence to decipher the paleoenvironmental and biogeochemical history of the past hydrothermal system at Santorini caldera.

## Abstract (web summary)

Santorini caldera hosted a vigorous, episodic hydrothermal system from ~1630 BCE to 726 CE, exceeding today’s activity. Geochemical and DNA evidence reveal major metal fluxes, microbial adaptation, and links between volcanism, hydrothermalism and submarine life.

## Introduction

Hydrothermal activity provides energy and material fluxes in volcanic settings, which are vital for chemosynthetic life, biogeochemical cycling and critical metal resources^1–3^. Hydrothermal activity also promotes rock alteration, modifying porosity and permeability^4^ and reducing the rock strength >10-fold, thereby compromising volcano stability^5^. Shallow (≤200 m water depth)^2^ and intermediate-depth (200–1500 m water depth)^6^ hydrothermal systems associated with marine volcanoes in subduction settings are chemically distinct from better-studied ocean ridge vents^1,7–9^ exhibiting a broad range of fluid compositions^1^. Since magmatic volatiles strongly influence fluids in arc settings^3,10^, studying hydrothermal systems in subduction zone environments can provide new insights into chemical cycling between the deep Earth and surface reservoirs^1,3,7^.

Changes in the activity at shallow-marine hydrothermal fields may signal volcanic unrest, based on short-term observations of changes in seawater appearance (turbidity and colour) immediately before eruption^11,12^. However, there are no long-term records (>20 years)^13–15^ of the behaviour of hydrothermal systems in restless, flooded calderas, where the deep and extensive magmatic system and readily available fluids may promote sustained subaqueous hydrothermal activity. Indeed, the long-term nature and behaviour of hydrothermal systems, particularly in arc settings, remains very poorly constrained. Apart from studies of the hydrothermal system at Brothers volcano in the Kermadec Arc^16^, there are few estimates of either longevity or variability of such systems through time^17^, resulting in uncertainties in the relationships between hydrothermal activity, volcanic events, metal fluxes and impacts on marine geochemical cycles^13,18^.

**Fig. 1:**
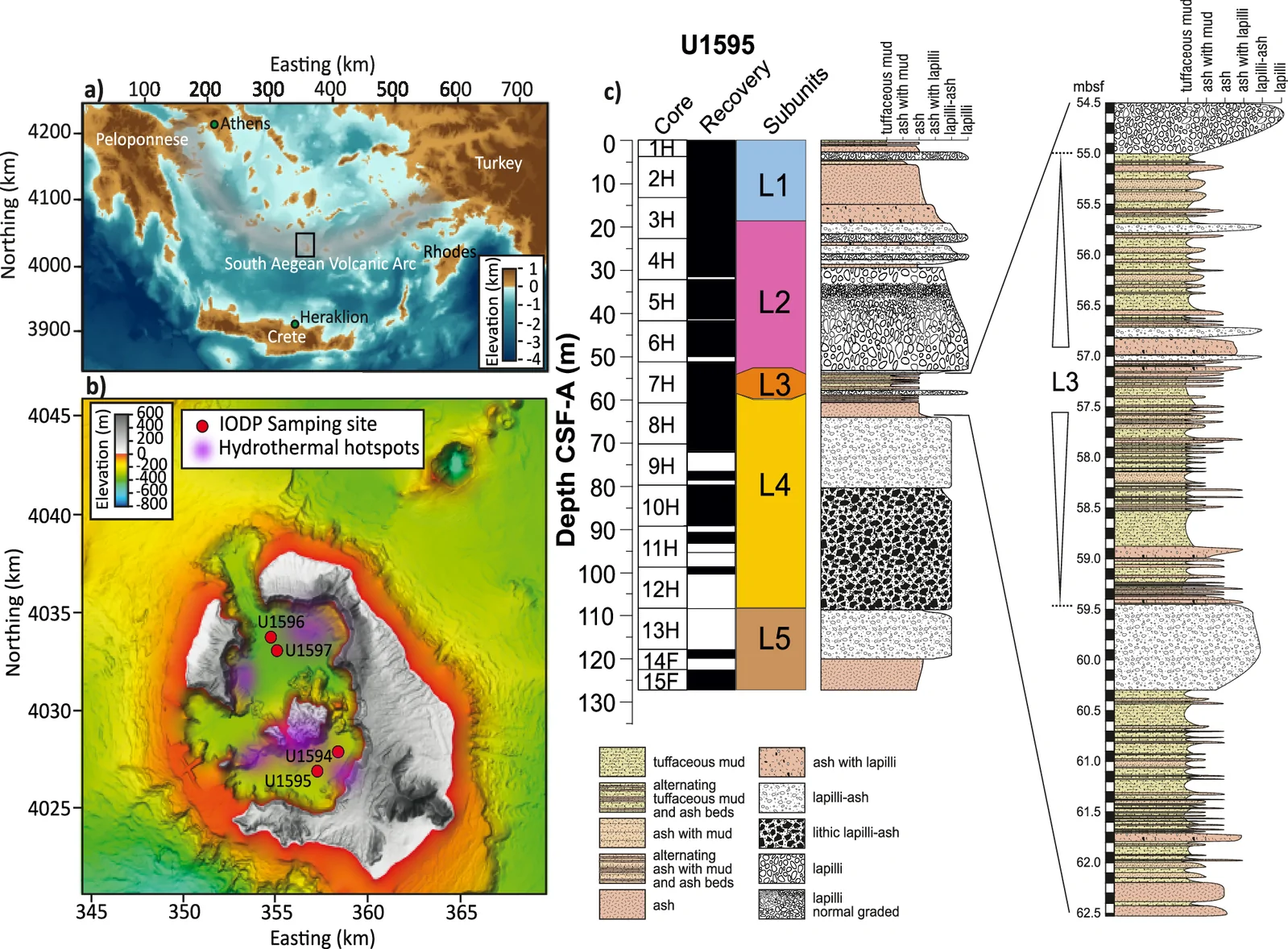
Geological area and lithostratigraphy. **a** Map of the South Aegean Volcanic Arc (shaded grey)^49^. The black rectangle highlights the location of Santorini caldera. The coordinate system here is UTM Zone 35 N, WGS84 datum. **b** Morphological map of the Santorini caldera^49^, showing the IODP Expedition 398 drill sites (red circles). Areas of currently known hydrothermal activity are shown in purple^32^. **c** Generalised lithostratigraphy of the intra-caldera deposits^49^. Depth of lithologic units L1–L5 with the corresponding core names, recovery and lithostratigraphic profiles for Site U1595. L1–L4 from Hole U1595A and L5 from Hole U1595B were used to form a complete stratigraphy. Black areas in the recovery column indicate core recovery, and white areas indicate recovery gaps.

The Santorini caldera, Greece (Fig. 1a), is a prime example of a marine-flooded caldera in a volcanic arc and offers a rare opportunity to address this knowledge gap^7,8^. The semi-enclosed nature of Santorini’s caldera promotes the preservation of the chemical signatures from hydrothermal fluids, when their elemental load is precipitated or scavenged and deposited on the caldera floor^1,3,19^.

The flooded caldera of Santorini (84.9 km^2^)^20^ was created during multiple large, explosive eruptions^21–23^, the latest being the Late Bronze Age Minoan eruption of *ca*. 1600–1630 BCE^24,25^. The caldera comprises three basins: a 390-m-deep northern, 320-m-deep southern and 290-m-deep western basin^19,26,27^, and holds *ca*. 27 km^3^ of seawater. The islets of Palea Kameni and Nea Kameni are the subaerial summits of a volcanic edifice that has grown within the caldera since the Minoan eruption^21,27,28^. For decades, active low-temperature, acidic (<35 °C, pH < 6) hydrothermal activity in shallow embayments around the Kameni Islands^26,28^ has enriched local sea-floor sediments with metals, including Cu, Pb, V, Fe, Mn, Mo, Hg, As and Sb^7,8,19,29^, and microbes have developed resistance to high concentrations of toxic metals^30,31^. Submersible surveys have found vent fields on the caldera floor, including multiple metre-scale bacterial mats and CO_2_ seeps^32,33^. Mercury (Hg) is a notable hydrothermal proxy^34–36^ in volcanic settings^37^ due to its large magnitude differences between background concentration in seawater (~0.4–3 pM for the Mediterranean Sea^38^) and hydrothermal fluids and associated sediments^34,35,39^. It is also considered to be geochemically stable in marine burial settings^40^ and easy to measure^41^. However, few studies have quantified marine hydrothermal Hg fluxes^42,43^, leaving the contribution from shallow venting to global geochemical cycles unresolved.

Investigation of the hydrothermal system in Santorini caldera found that fluids sampled between 1982 and 1997 from the Kameni Island embayments exhibited, on average, a stable chemical composition^26^. Compared with other hydrothermally-active centres along the South Aegean Volcanic Arc, these fluids were the most concentrated in Mn and Fe, reaching a thousand-fold enrichment over normal seawater^7,26^. While there is little evidence for variation on decadal time-scales during a period of volcanic quiescence, analysis of records over longer time-scales is essential to understand the potential links between deeper magmatic supply, volcanic unrest or eruption and changes in hydrothermal activity.

Drilling of Palea Kameni in 1988 recovered 200 m of oxidised lavas and volcanic sediments, but did not reach any areas of epithermal alteration^44^. Previous work within the Santorini caldera has been restricted to sampling the youngest sea-floor sediments from short cores (~60‒250 cm)^19,45^. These analyses showed that caldera sediments were influenced by hydrothermal activity, since mean concentrations of Mn, Fe, Cu, Zn, Pb and P were higher in intracaldera sediments than outside the caldera. The cores contained distinct Mn and Fe-enriched layers at depth, which were considered to represent a hydrothermal past that was considered ‘more active’ than the present-day, whether from higher-temperature venting, carrying more metals to the seafloor, or an increase in active vents within the caldera. To date, no hydrothermal fluids from the basin floors have been analysed on Santorini.

Submarine hydrothermal systems also provide a distinctive environment for microbial communities, and microbial activity is known to play an essential role in the cycling of iron in the shallow hydrothermal embayments^46,47^ around the Kameni Islands^31^. Microorganisms can act as sensitive recorders of environmental conditions, and may directly influence the fate of hydrothermally derived elements^2^. Thus, understanding how microbial communities respond to hydrothermal perturbations improves our ability to interpret marine, seafloor and subsurface biogeochemical processes.

International Ocean Discovery Program (IODP) Expedition 398 deep-drilled the seafloor sediments of the Santorini caldera^48^ in 2022/2023. The volcano-sedimentary sequence that was recovered provides a continuous geological archive spanning the last ~3500 years, including the deposits of a major submarine eruption (726 CE eruption)^49^ and allows us to track the trace metal and microbial signatures of hydrothermal activity within the Santorini caldera over this same time period. Here, we analyse the sedimentary archives, with supporting microbial evidence, to resolve past hydrothermal activity at Santorini.

## Results and discussion

### Lithologies

IODP Expedition 398 cored four sites in the northern (sites U1596 and U1597) and southern (sites U1594 and U1595) caldera basins (Fig. 1b). Five units (L1–L5) were described shipboard^48,49^, all of which were unlithified. Units L1 and L2, consisting of pumice and ash from historical volcanic activity and the 726 CE eruption, respectively, were partly recovered at all sites, while L3–L5, comprising volcanic and non-volcanic material, were only recovered at Site U1595. Hence, Site U1595, consisting of three closely-spaced holes (U1595A–C), is the focus of our analysis (Fig. 1c). The acoustic basement, thought to be eruptive products of the ~1630 BCE Minoan eruption^49^, was not penetrated^48,49^. In Hole U1595A, Unit L1 extends to ~20 metres below seafloor (mbsf) and comprises well-sorted ash and lapilli-ash. Unit L2 (~20–55 mbsf) consists of well-sorted pumice and lapilli-ash from the 726 CE eruption^49^. Unit L3 (~55–59.5 mbsf) is the most lithologically variable unit with intercalated ash, tuffaceous mud and green-brown-red coloured banding, indicating a different depositional environment to other units. Unit L4 (~60–110 mbsf) contains heterolithologic sands and gravel. Unit L5 (only recovered in Hole U1595B, below *ca*. 110 mbsf) reached 126 mbsf (the limit of penetration), featuring red-hued pumice and lithic layers. Cores were sampled every 20 cm across Units L1–L4 (U1595A) and L5 (U1595B). Parts of Unit L3 were sampled at 1-cm resolution to resolve variability in elemental composition associated with the coloured banding and differing lithologies. This material from IODP Expedition 398 is the first deep (>2.5 mbsf) sediment analysed from inside Santorini caldera and offers unique insights into the past caldera-floor conditions during and between the known eruptions of the Kameni Islands over the last ~3500 years.

### Timescales

We have estimated sedimentation rates for the deposits using the 726 CE eruption at Site U1595 (southern basin) as a timestamp. Since the eruption can be considered an instantaneous event, we denote the top of the 726 CE pumice (Unit L2) as the year 726 CE. This allows us to calculate an average sedimentation rate for L1 between 726 CE and the time of drilling in 2023 CE. We exclude any instantaneous deposits (tephras) from the rate estimate and assume that ‘tuffaceous mud’ represents the background sedimentation within the caldera (Fig. 1). Hole U1595A contains 1.58 m of tuffaceous mud deposited between 726 CE and 2023 CE (Unit L1), corresponding to a sedimentation rate of 0.122 cm yr^−1^. We then use this rate to determine the timescales of the other lithological units, assuming the same first-order sedimentation rate. In Unit L3, there is 2.77 m of tuffaceous mud, which suggests this unit spans ~2270 years. This implies that the base of Unit L3 dates from ~3570 years ago, i.e. directly after the Minoan eruption of the 17^th^ century BCE. This requires that Units L4 and L5, the volcanic and lithic material, were deposited very rapidly after the caldera-forming eruption^50^.

### The hydrothermal past of Santorini

Concentrations of selected trace elements, considered likely to be associated with hydrothermal activity^51^ (i.e. Hg, Sb, As, Mn, Mo, V, Fe, Co, Cu, Pb, Ag), were analysed in bulk samples of the unconsolidated and unmineralized sediments from Holes U1595A (L1–L4) and U1595B (L5). Many of these elements show elevated concentrations in lithological Unit L3 (Supplementary Fig. 1).

**Fig. 2:**
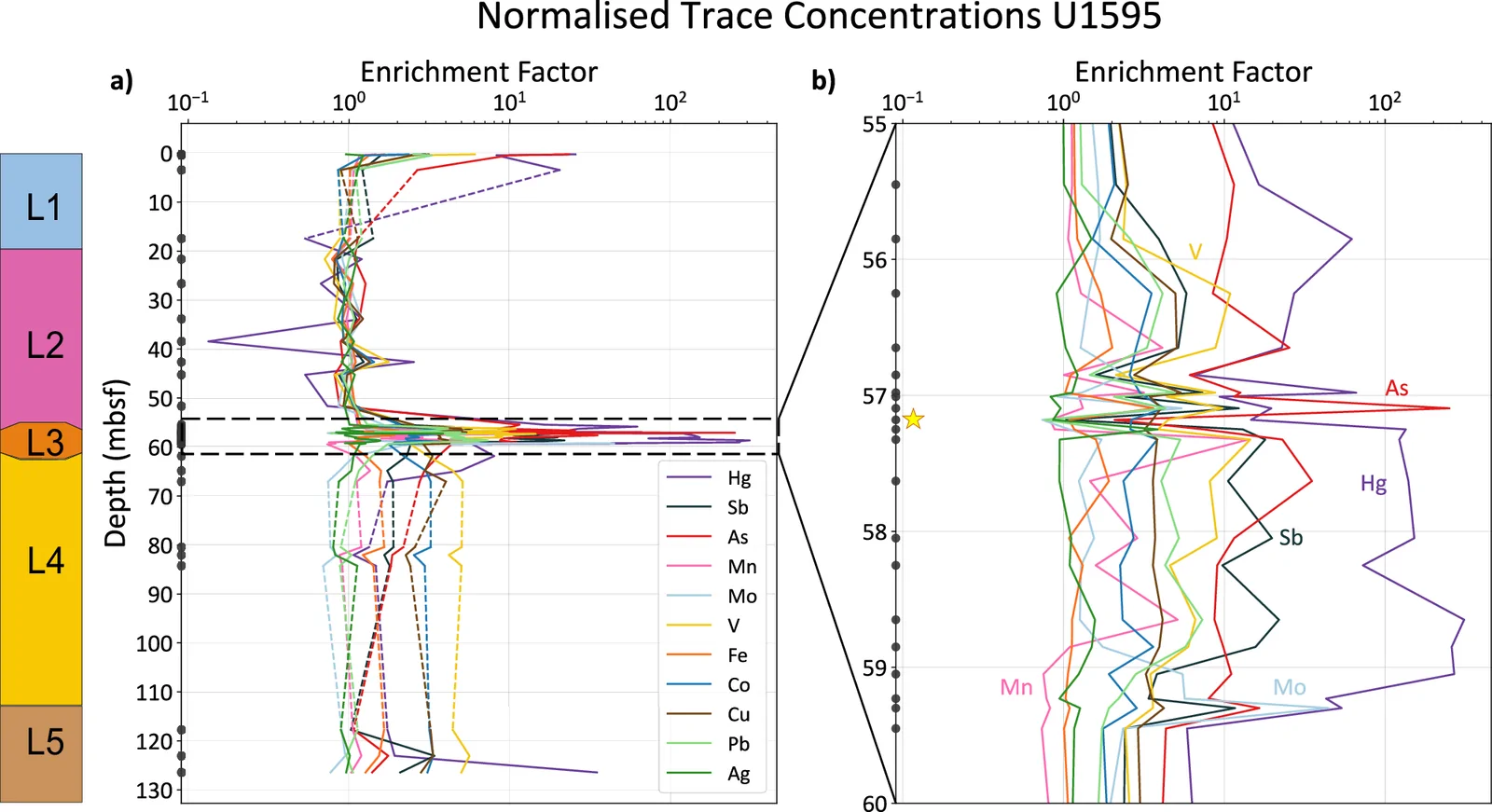
Normalised trace element concentrations for site U1595. Overall lithostratigraphy for Hole U1595A is displayed to the left. Note the x-axes are on a log scale, and sampling point depths are shown as black circles on the y-axes. **a** The whole sequence showing the full depth of the site and the element enrichments, 0–90 mbsf from Hole U1595A and 108–127 mbsf from Hole U1595B. Dashed portions of the lines indicate breaks in sampling resolution. **b** A zoomed-in section covering depths 55–60 mbsf (Unit L3), to be able to see in detail large enrichments at those depths in Hole U1595A. Yellow star denotes location of microbiology sample—note microbiology sample was taken from Hole U1595C so it has been depth matched onto Hole U1595A here.

All sample concentrations were normalised to 726 CE (Unit L2) mean values (see ‘Methods’), to obtain enrichment factors (EF) relative to volcanic ‘background’ (Fig. 2). We find a >200-fold enrichment for both As and Hg at 57.095 and 58.65–59.05 mbsf, respectively. Significant enrichments are also found for Mo (EF 43, 59.3 mbsf) and Sb (EF 22, 58.65 mbsf), whilst V and Mn yield EFs of 14 (57.325 mbsf). Copper, Pb, Fe, Co and Ag all peak at EF < 10, with the maximum enrichments of Co and Fe aligning with that of As (at 57.095 mbsf), and the maximum in Pb aligning with Hg at 58.65 mbsf.

Though significant metal enrichments occur predominantly in Unit L3, near-surface sediments at the top of Unit L1 are also enriched in most of the trace metals, namely Hg and As (Fig. 2). Arsenic, Sb and Hg are typically enriched in arc volcano hydrothermal fluids and gases, and often associated with epithermal deposits^52,53^.

Previous studies on near-surface sediments from Santorini caldera reported similar elevated metal concentrations of Mn, Fe, Co, Cu, Zn, Pb and Hg within the top metre of sediment^7,19^ (Table 1) compared to samples taken from outside the caldera^54^. Arsenic and Sb have been measured in the hydrothermal sediments of hot-spring embayments of the Kameni Islands, with values of 927 ppm As and 3.21 ppm Sb^29^. In Unit L3 at Site U1595, we find maximum values of 1030 ppm As and 4.8 ppm Sb, higher than active hydrothermal sediments in the present-day caldera.

**Table 1:** Comparison of element concentrations (in ppm) for bulk surface sediments from literature with our near-surface sample from Hole U1595A and sediments outside the caldera

	Mn	Fe	Co	Cu	Zn	Pb	Hg
Southern basin sediments, 25 cm depth (this work)	1400	47400	10.7	30.6	85	31.9	0.0192
Southern basin sediments, L3 maximum (this work)	13500	104000	18	52.6	132	92.5	0.233
Average of core-top sediments from two southern basin cores (Cronan et al.^19^)	1870	42200	9.5	24.5	85.5	36	n.d.
Sediment from southern basin core (Smith and Cronan^7^)	1600	42000	8	30	87	23	0.037
Sediments from outside the caldera (Hodkinson et al.^54^)	641	18700	9	19	39	17	n.d.

The maximum values for each element within Unit L3 are also included here for further comparison. Only values from other caldera sediments are included, not samples from near-shore the Kameni Islands. Non-normalised values.

n.d. not determined.

Our new data show a distinct enrichment in hydrothermally-derived trace metals between 55 and 60 mbsf (Fig. 2, Table 1), below the pumice deposits from the explosive eruption of 726 CE. This enrichment is over ten times larger than anomalies seen at the seafloor surface today in the same core (Fig. 2a). We infer that this considerable metal enrichment was due to previously unknown submarine hydrothermal activity, that was more vigorous than the present-day and which persisted for much of the time between the 1650 BCE Minoan eruption and the 726 CE eruption, but which also shows variations in vigour through time. We suggest that the variations in the metal enrichments that we see reflect changing fluxes of hydrothermal metal inputs into the caldera, and provide compelling evidence for the prolonged activity of Santorini’s submarine paleo-hydrothermal system.

### Genomic microbial signatures of hydrothermal metals

We successfully obtained DNA from live organisms from one sample collected from Unit L3 at Hole U1595C (55 mbsf, equivalent to 57.25 mbsf at Hole U1595A, Fig. 2b; ‘Methods’). Recovering high molecular weight DNA from deep subsurface sediments is a challenging task due to low biomass and DNA degradation with depth and time. Shotgun metagenomic sequencing enabled screening for genes involved in metal resistance and redox transformations. This can help constrain the seafloor conditions at the time of deposition during past hydrothermal events.

**Fig. 3:**
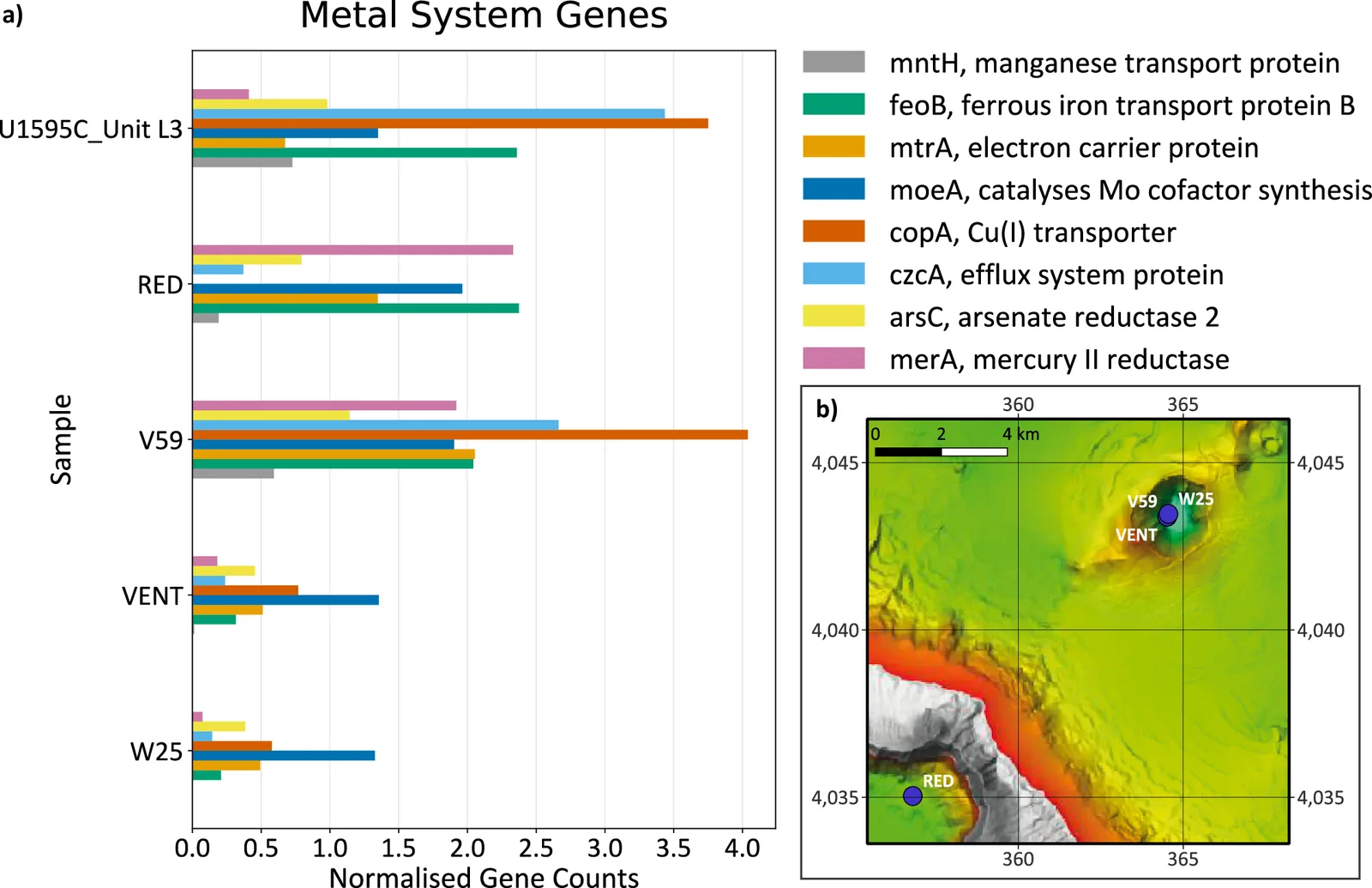
Metal system genes. **a** Normalised gene counts of key metal resistance, transport and redox-related genes in the Unit L3 sample from Hole U1595C, compared with the reference hydrothermal vent samples RED, V59, the distal vent sample VENT and the non-hydrothermal sample W25. **b** Map indicating sample locations for reference samples, the coordinate system here is UTM Zone 35 N, WGS84 datum.

The results reveal a diverse repertoire of genes encoding proteins responsible for resistance to toxic metals. Most metals are essential to the function of organisms, but in elevated concentrations, they can disrupt homoeostasis and become toxic for marine microorganisms. We identified systems offering resistance or adaptation to elevated levels of Hg, As, Cu, Mn, Fe, Mo, Co, Zn and Cd. These mechanisms are key for element cycling in metal-enriched environments. Notably, the *mer*A gene from the Hg resistance system, coding for the mercury II reductase protein, which converts organic-bound Hg^2+^ to volatile Hg^0^, was present^30,55^ (Fig. 3a and Supplementary Fig. 2). High levels of *mer*A is consistent with a selective pressure from dissolved Hg^2+55^. Similarly, arsenate reductase genes (*ars*C)^56^, copper resistance and efflux genes (*cop*A, *cus*A, *cus*B)^57^, and the Co/Zn/Cd efflux system (*czc*A-D)^58^ all selectively remove or detoxify the metal they are specific for due to increased exposure from those metals. The Fe^2+^ transporter (*feo*B) imports ferrous iron under reducing or low pH conditions^59^ and the *mtr* system instead transfers electrons to extracellular Fe^3+^^60^. The Mn^2+^ transporter (*mnt*H) imports Mn^2+^ when it is scarce; high levels of *mnt*H imply fluctuating redox conditions^61^. Finally, the Mo cofactor biosynthesis is regulated by the *moe*A gene, without it, Mo would not be incorporated into the cell^62^. For full normalised gene counts for these systems see Supplementary Fig. 2.

We compare the normalised gene counts (see ‘Methods’) of Unit L3 with those from presently hydrothermally active and inactive areas. Samples RED (Santorini caldera seafloor) and V59 (hydrothermal vent chimney of Kolumbo volcano, 7 km NE of Santorini) represent contemporary hydrothermal environments. Sample VENT is distal vent water above V59, and W25 is normal seawater, chosen to represent a non-hydrothermal environment (Fig. 3b).

We taxonomically assigned the metal resistance genes to archaeal and bacterial phyla (see Supplementary Fig. 3). These data support the interpretation that Unit L3 formed during metal-enriched hydrothermal conditions. In particular, high abundances of *czc*A (3.44), *cop*A (3.75), *mnt*H (0.727) and *feo*B (2.36), along with elevated levels of *ars*C (0.98) and *mer*A (0.41) in Site U1595 compared to the W25 background (0.14, 0.58, 0.0, 0.21, 0.38, 0.07, respectively) (Fig. 3a) are consistent with a prolonged exposure to metal-rich hydrothermal activity. The prevalence of efflux systems (*czc* and *cop*) implies a strong selective pressure for detoxification and intracellular metal homoeostasis. The low levels of *mtr*A genes (0.67 and 0.49 at Site U1595 and in the W25 background, respectively) indicate that Fe^3+^ reduction was not a dominant pathway, potentially due to the fact that Fe was mostly present as dissolved Fe^2+^ in hydrothermal fluids rather than particulate Fe^3+^ oxides. This is supported by the increased *feo* levels, which suggest bioavailable Fe^2+^ under reducing conditions—typical of hydrothermally influenced sediments^63^. Sediment concentrations of Mn (mobile under reducing conditions) and Mo (immobile under sulfidic conditions) peak at different depths in the L3 sediments (Fig. 2b), providing further evidence for reducing conditions and redox fluctuations. Collectively, this gene distribution reflects the nature of the fluids and sediments at the time of deposition, and supports the interpretation of the hydrothermal origin of the trace metals in Unit L3. In particular, we note that Unit L3 showed higher gene abundances for the *czc, mnt* and *feo* systems than in the chimney sample V59, which comes from a high-temperature, low-pH, vigorous hydrothermal system in Kolumbo volcano^51,64^. These gene elevations indicate a higher metal flux in Santorini’s past than we see in the present-day caldera, perhaps with metal fluxes and hydrothermal activity similar to present-day Kolumbo volcano.

### Evolution of the pre-726 CE hydrothermal system

**Fig. 4:**
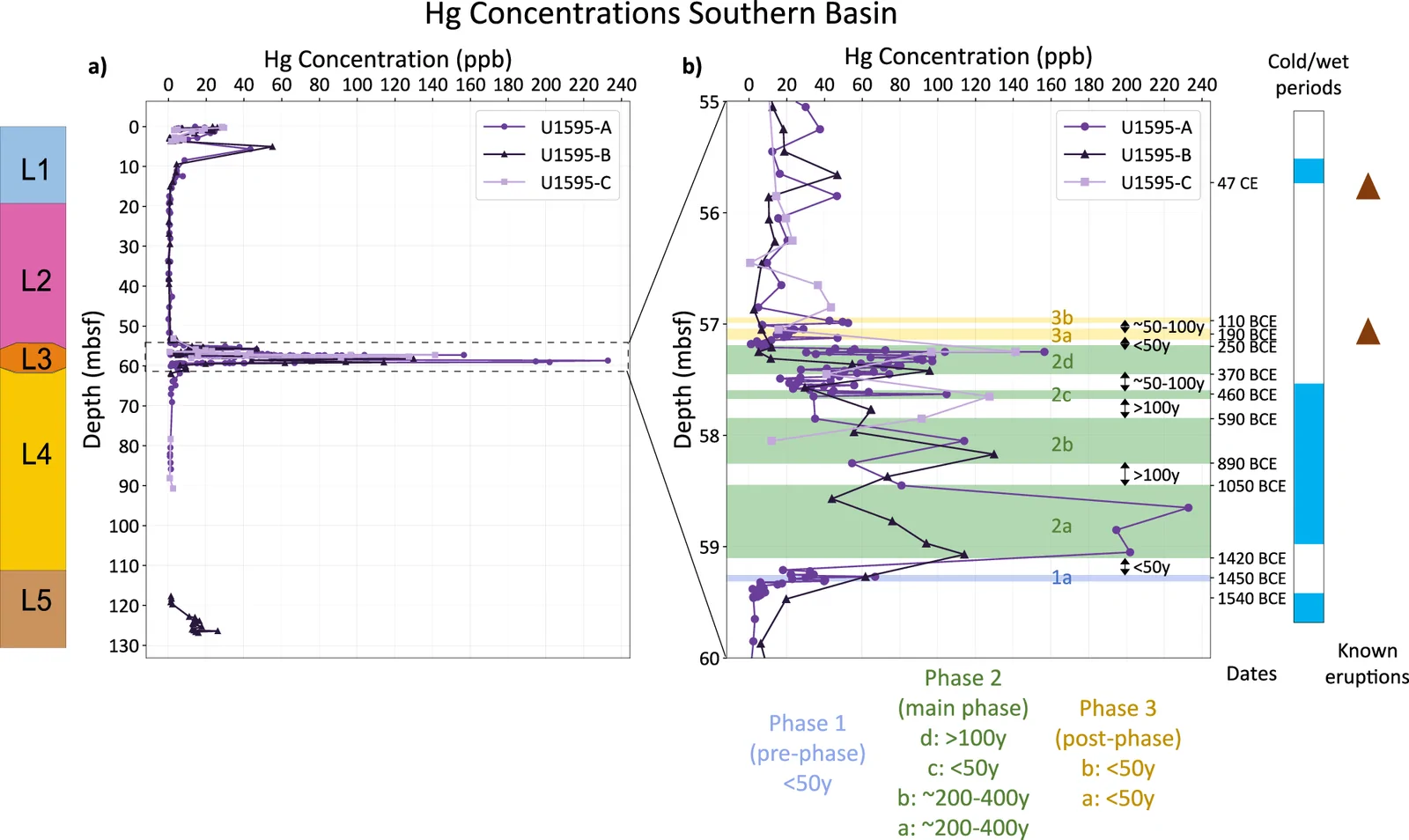
Hg concentrations down-core at Site U1595 with phased hydrothermal activity. **a** Hg concentrations for each hole: U1595A, U1595B, U1595C from seafloor to ~126 mbsf (maximum depth). Breaks in the line indicate sampling and core recovery gaps. **b** Depth interval encompassing Unit L3 (55–60 mbsf), with the phases/pulses of hydrothermal activity highlighted. The phases are colour-coded according to the type of phase: pre-phase, main phase or post-phase. The phases have been split into sub-phases labelled a–d. Estimated durations of phases are written in the key below the graph, with the duration of hydrothermal quiescence written in black and labelled with double-headed arrows directly on the graph. Approximate dates have been assigned to the right-hand y-axis. Cold/wet climate periods are highlighted in blue in the right-hand bar. The brown triangles represent the known eruptions of the Palea Kameni Island (197 BCE and 46–47 CE). To ensure a direct comparison, data from Holes U1595B and U1595C were aligned with U1595A using the upper boundary of Unit L3. Uncorrected graph can be found at Supplementary Fig. 4.

The metal enrichments in the sediments over the 2300-year period recorded by Unit L3 are both elevated above present-day levels and are variable. This could indicate pulsing of hydrothermal activity or differing extents of sediment dilution, resulting in the time-varying patterns of the observed elemental enrichments. By analysing the tuffaceous mud layers, we can map out the nature of Santorini’s past intervals of hydrothermal metal enrichment and use our sedimentation rate estimates to approximate their durations (Fig. 4). We combined the Hg data from the three Holes at Site U1595 and focus on this high-resolution composite record to construct a detailed record of hydrothermal phases (Fig. 4a). The stratigraphic interval of elevated Hg in Unit L3 (55–60 mbsf) can be divided into three phases of metal enrichment (Fig. 4b), and we can estimate timescales of each of these phases using the tuffaceous mud sedimentation rate (0.122 cm yr^−1^). On this basis, Phase 1 (pre-phase) lasts <50 years and shows a maximum Hg ~60 ppb, Phase 2 (main phase) collectively lasts 500–1000 years, and has Hg >80 ppb; while Phase 3 (post-phase) lasts 50–100 years with Hg between 40 and 60 ppb. Altogether, this implies a maximum of ~1100 years of heightened hydrothermal activity during the 2300 years of Unit L3. The ranges in timescales reflect irregularity in sample resolution, and we estimate uncertainties to the nearest 10 years (Supplementary Table 1).

We compare the approximate timeline of the inferred hydrothermal pulses, along with known eruptions of the Kameni Islands^27^, and the regional (Aegean) climatology^65^ (Fig. 4b). Most of the strongest episodes of hydrothermal activity (Phase 2) fall within an extended climatologically cold/wet period^65^ and terminate around the time of the 197 BCE eruption, which corresponds to the emergence of present-day Palea Kameni. There may be another pulse of hydrothermal activity corresponding with the eruption in 46–47 CE, which also occurs during a cold/wet period, although this inference is only supported by a single-point increase in Hg. The roles of the hydraulic head (groundwater) and edifice loading (Palea Kameni) in modulating the hydrothermal fluxes require further investigation. We speculate that the growth of the Kameni edifice may have helped to seal the hydrothermal system in the lead up to the explosive submarine eruption of 726 CE, since we observe a notable decrease in Hg enrichments at ~57 mbsf (beginning of post-caldera volcanic activity, in 197 BCE) and in Unit L1 (post-726 CE) (Fig. 4).

Within the present-day caldera, there are no known sea-floor hydrothermal vents close to the U1595 coring site. Instead, the closest vents are ~2.5 km away (Fig. 1b) and appear to be constrained to specific areas around the caldera ring faults or the present-day Kameni edifice. We assume that this spatial pattern was established following the ~1600 BCE Minoan eruption. Distal seafloor sites can give basin-wide information about the influence of hydrothermal inputs into the flooded caldera, as the metal-rich hydrothermal plumes rise from the submarine vents, entrain and mix with ambient seawater, and their metals are scavenged by Fe and Mn oxides^37^. Co-enrichment of Hg with Fe and Mn is indicative of a hydrothermal plume settling out into the sediments, which is consistent with our data (Fig. 2 and Fig. 4). In both the Central Indian Ridge and Kagoshima Bay in Japan, hydrothermal Hg was detected 1–10 km away from active vent sites^36,37^. Surface sediments proximal to the hydrothermal field in Paleochori Bay, Milos (neighbouring island to Santorini), show Hg concentrations of ~200 ppb, similar to our distal sediments prior to the 197 BCE eruption of Santorini (Fig. 4b).

Short (<20 years) time-series at other volcanic systems have alluded to the nature of hydrothermal system evolution^13–15,66^, with slow-spreading ridges remaining stable over time^66^ and volcanically active arc settings exhibiting dynamic behaviour^15^. At Brothers volcano in the Kermadec Arc, dated chimney fragments extend back 1100 years^16^, pointing to the longevity of a hydrothermal system. The work we report here tracks the continuous behaviour of a hydrothermal system over two millennia, and corroborates that hydrothermal systems in volcanically active environments are not stable, but may wax and wane^16^ over decadal to centennial timescales, at least in terms of their surface outputs.

### Metal Budgets and Fluxes

From the known trace metal concentrations associated with the Unit L3 hydrothermal anomaly, we can estimate the element budgets captured in the sediments of the southern basin and the whole caldera:

1 $$m\,=\,z\,\times \,\rho \,\times \,C\,\times \,A$$

where *m* is the ‘excess’ mass of the element of interest (mg) compared to non-hydrothermally affected deposits (e.g. Unit L2, the 726 CE eruptive deposit), *z* is the tuffaceous mud sediment layer thickness (2.77 m for L3), *ρ* is the sediment dry bulk density (967 kg m^−3^ for tuffaceous mud in Unit L3, measured shipboard), *C* is the element concentration anomaly over background (ppm), and *A* is the area of the anomaly, assuming vents deposit distally within the caldera^19^ (1.8 × 10^7^ m^2^ for the southern basin and 8.5 $\times \,$10^7^ m^2^ for the whole caldera). While we only have chemical analysis for the Site U1595 core material, Unit L3 is present in seismic-reflection profiles across the entire caldera^49^.

For Hg, the excess metal budget above background (*m*) is ~3000  kg for the southern basin and ~14,000 kg for the entire caldera (Table 2) over the 2270-year timespan of Unit L3. We can also determine a metal budget for each phase of Hg enrichment (Table 2). These metal budgets can be compared with those of the post-726 CE hydrothermal system (Unit L1). Since 726 CE, 1.58 m of tuffaceous mud sediment was deposited (average dry bulk density of 699 kg m^−3^) in the caldera, and with an excess mass of deposited Hg of 470 kg (southern basin) to 2200 kg (entire caldera).

**Table 2:** The Hg budgets and depositional fluxes (excess over background) for each phase of hydrothermal activity within Unit L3 (Fig. 4), all of Unit L3 and post-726 CE (Unit L1) venting scaled to the southern basin and the whole caldera

	southern basin		caldera	
Phase	Hg Budget (kg)	Hg Flux (kg yr^−1^)	Hg Budget (kg)	Hg Flux (kg yr^−1^)
Post-726 CE (Unit L1)	467	0.4	2206	1.7
3b	16.5	0.8	78.1	3.9
3a	13.1	0.7	61.6	3.1
2d	174	1.5	820	6.8
2c	24.3	1.2	115	5.7
2b	729	2.4	3444	11.5
2a	1385	3.7	6539	17.8
1	20.5	0.7	96.7	3.2
Pre-726 CE (Unit L3)	2982	2.2	14080	10.1

The Unit L3 values are calculated independently of the phases by calculating an average Hg value for all tuffaceous mud layers outside the phases, then the Entire Unit L3 values are the result of adding all the phase values onto that average. The timescales for the phases used to calculate the fluxes can be found in Supplementary Table 1. For Unit L3, the timescale is 2270 years, and for the post-726 CE system, it is 1297 years.

In Table 2, we estimate the annual Hg depositional fluxes for each phase of hydrothermal activity between the Minoan and 726 CE eruptions. The average hydrothermal Hg deposition flux was 10 kg yr^−1^ for the whole of Unit L3, and *ca*. 18 kg yr^−1^ during hydrothermal phase 2a. These values are minima for the hydrothermal Hg fluxes from the system at the time, since not all of the hydrothermally-sourced Hg may have been deposited in the caldera sediments. However, in a similar volcanic setting, Kagoshima Bay, Japan (a 200 m deep enclosed basin with a seafloor hydrothermal system), little hydrothermal Hg is found in surface waters, suggesting approximately complete deposition within the basin^67^.

Our measurements show that Hg fluxes from the pre-726 CE hydrothermal system were significantly greater than those since 726 CE. The time-averaged Hg flux across Unit L3 is 6× greater than the modern-day (Unit L1) hydrothermal system; while the flux for Phase 2a was an order of magnitude larger (Table 2). Although we might expect to see the influence of anthropogenic Hg after 1850 CE^68^, this contribution is in fact quite small. We can estimate the relevant anthropogenic Hg flux from regional (Ionian Sea) background marine sediment Hg accumulation rates. For the Ionian Sea post-726 CE, the average sedimentary Hg accumulation rate is 1.1 µg m^−2^ yr^−1^^68^, equivalent to a whole-caldera Hg flux of 0.09 kg yr^−1^, or ~5% of our post-726 CE values. This indicates that Hg depositional fluxes in the Santorini caldera are dominated by hydrothermal inputs throughout the entire record.

The only other submarine Hg flux that has been determined for a subduction zone hydrothermal system is in the Tonga Arc^42^, which yielded a total hydrothermal Hg emission flux of 35 kg yr^−1^, about ~3× higher than the average for Santorini’s hydrothermal system prior to 726 CE (Table 2, entire caldera). For a mid-ocean ridge setting, the annual Hg flux from the Trans-Atlantic Geotraverse (TAG) in the Mid-Atlantic Ridge is estimated at a maximum of 4.5 kg yr^−1^, which is comparable with our flux ranges at Santorini (if at the lower end of the entire caldera-based fluxes). However, both the Tonga and TAG values were calculated from hydrothermal fluids and plumes themselves, whilst we are estimating the minimum Hg flux from analysis of distal sediments alone. Future analysis of hydrothermal fluids or of proximal sediments would be useful next steps in completing an Hg flux estimate for Santorini and understanding its impact on biogeochemical cycles. While the true hydrothermal Hg emission flux from Santorini is not yet confirmed, this helps to constrain the potential scale of such emissions globally. Based on our limited data, we suggest that hydrothermal systems in subduction zones are significant point sources of Hg release to the oceans.

We see similar temporal trends in other hydrothermally-derived trace metals (Table 3) with Mn, V, Co, Cu, As, Mo, Sb, Pb and Ag all exhibiting fluxes pre-726 CE (Unit L3) which were an order of magnitude larger than post-726 CE (Unit L1). The quantities of metals deposited within the caldera-floor sediments during the pre-726 CE hydrothermal event included 5900 tonnes of Cu, 1620 tonnes of Mo and 16 tonnes of Ag. The deposition fluxes of Ag, Hg, Sb and As that we estimate for Unit L3 are similar to the hydrothermal fluid fluxes estimated for the present-day Broadlands-Ohaaki and Rotokawa geothermal fields in Taupo, New Zealand^10^. This comparison confirms that submarine arc volcanoes can also be significant sources of hydrothermal metal enrichment, and of metal emission to the hydrosphere and atmosphere. By analogy with the Taupo volcanic system, we would expect that the sustained release of metals from Santorini hydrothermal vents since the caldera-forming eruption 3500 years ago is the sea-floor expression of a shallow crustal epithermal system, which has the potential to concentrate and deposit metals.

**Table 3:** Metal budgets in Unit L3 (pre-726 CE hydrothermal system) and Unit L1 (post-726 CE hydrothermal system) for Santorini’s southern basin and the whole caldera for the most enriched elements in IODP Expedition 398 cores

	*Unit L1 southern basin*		*Unit L1 caldera*		*Unit L3 southern basin*		*Unit L3 caldera*	
	Budgets (t)	Fluxes (t yr^−1^)	Budgets (t)	Fluxes (t yr^−1^)	Budgets (t)	Fluxes (t yr^−1^)	Budgets (t)	Fluxes (t yr^−1^)
*Mn*	3390	2.61	15800	12.2	58600	25.8	274000	121
*Fe (kt)*	144	0.111	670	0.517	548	0.241	2560	1.13
*V*	866	0.668	4040	3.11	6570	2.89	30600	13.5
*Co*	31.4	0.0242	147	0.113	379	0.167	1770	0.780
*Cu*	175	0.135	817	0.630	1260	0.555	5890	2.59
*As*	670	0.517	3130	2.41	4570	2.01	21300	9.38
*Mo*	33.0	0.0254	154	0.119	347	0.153	1620	0.714
*Sb*	3.42	0.00263	15.9	0.0123	79.4	0.0350	371	0.163
*Pb*	256	0.198	1200	0.922	1440	0.634	6740	2.97
*Ag*	0.335	0.000258	1.56	0.00121	3.44	0.00152	16.1	0.00709

Concentrations are averages across the whole unit and sediment thickness based on the tuffaceous mud layers. All budget values are in metric tonnes and fluxes in tonnes per year, except for Fe, whose values are in kilotonnes (kt) and kt per year.

**Fig. 5:**
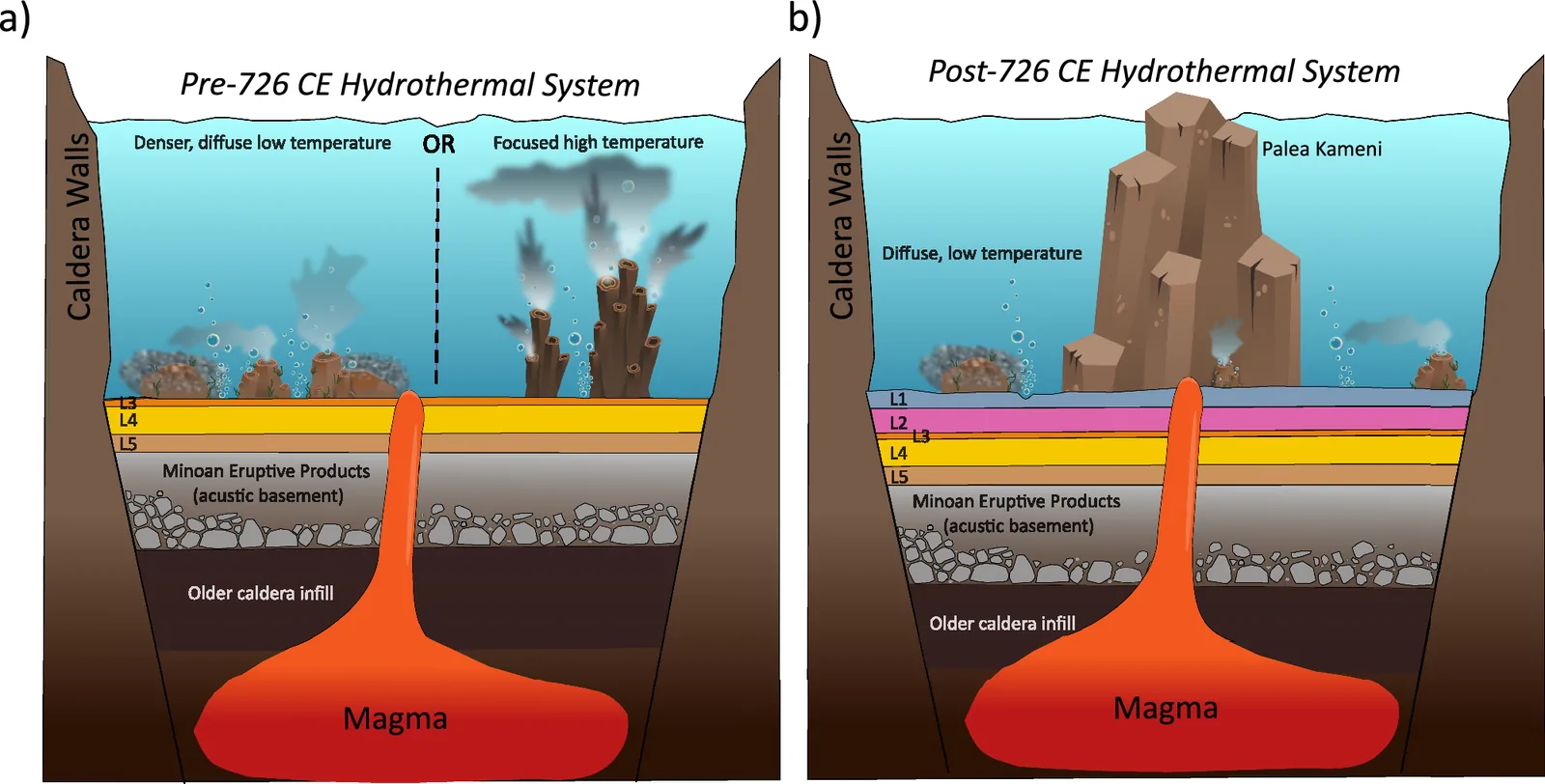
Conceptual diagram of Santorini’s hydrothermal system before and after the 726 CE eruption. An overview sequence of the caldera infill is shown for both the modern-day, with all lithological units found in Site U1595 shown, and for the pre-726 CE hydrothermal system that was active during deposition of Unit L3 between the Minoan eruption and the 726 CE eruption. **a** Prior to 726 CE, Palea Kameni island was beginning to form. Potential scenarios for the pre-726 CE hydrothermal system include a low temperature, diffuse system similar to post-726 CE, but with larger coverage and output across the caldera or more focused, higher temperature venting, able to produce increased fluxes compared to the modern-day. **b** The hydrothermal system in Santorini following the 726 CE eruption. Nea Kameni is not depicted as it began growing 800 years after the 726 CE eruption. This is the type of activity that is present in the caldera today: low temperature, mainly diffuse venting, with relatively low coverage across the seafloor. Objects in the image are not to scale.

Our deposition flux estimates for Fe and Mn for the past ~400 years within Unit L1 for Santorini’s southern basin are 111 t yr^−1^ (Fe) and 2.61 t yr^−1^ (Mn); these compare well with earlier estimates^45^ (88.9 t yr^−1^ Fe and 3.85 t yr^−1^ Mn, adjusted for measured bulk sediment density). The key observation is that elevated hydrothermal fluxes of the Santorini caldera were significantly elevated from *ca*. 1400 BCE to 110 BCE, compared to the modern-day at the same site, suggesting a larger hydrothermal source in the past than exists today. This is also supported by the elevated levels of metal resistance genes found in microbes in Unit L3 and evidenced by the smaller peaks and enrichments in Hg and trace metals in the shallow Unit 1 of the cores (Fig. 2 and Fig. 4) compared to Unit L3. A larger hydrothermal source could have resulted either from more widespread low-temperature, diffuse vents (as for the present day)^7,8,19,26,32^ across the caldera, or by higher-temperature flow transporting more metals to the seafloor^36,37,42^ (Fig. 5). Whether a hydrothermal system exhibits venting more characterised by one end member or the other will be controlled by a variety of factors such as permeability, seawater entrainment and magma supply^16^. Since Site U1595 was the only IODP drilling site that reached 55 mbsf, we are unable to analyse other caldera sites to distinguish between these hypotheses by quantifying the extent of the hydrothermal signal. However, since we see gene abundances for the Mn transport and Co/Zn/Cd efflux system surpassing the levels found in both present-day Santorini and at Kolumbo, which is a vigorous, high-temperature system, it is likely that the pre-726 CE hydrothermal system in Santorini was driven by focused, high-temperature activity, similar to that of present-day Kolumbo.

Our high-resolution sampling reveals the pulsatory nature of the ancient hydrothermal system, on timescales of decades to centuries. The parallel enrichment of hydrothermal metals and microbial adaptations demonstrate that hydrothermal activity was sufficiently intense and persistent. However, the limited present-day fluid data from the Santorini hydrothermal system restricts our capacity to constrain the past hydrothermal fluid emission fluxes and emphasises the need to integrate modern fluid data with past geological records to expand our understanding of the evolution of hydrothermal systems, the microbes that live there and their links to the volcanic system.

The episodic nature of the ancient hydrothermal system of Santorini could have had profound effects on local biogeochemical cycles for sporadic but extended periods, the effects and timescales of which remain unknown. Our results indicate that flooded calderas not only preserve evidence of past hydrothermal activity but may also act as long-term sinks for hydrothermally-derived trace metals. This accumulation of metal-enriched sediments illustrates the potential to form metal reservoirs that could be remobilised during later epithermal or acid-sulphate processes. By providing a long-term insight into the evolution of a past hydrothermal system and how it compares to its modern-day counterpart, this study sheds light on the evolving relationships between volcanic processes, hydrothermal systems, the local microbial environment and their metal bounty.

## Methods

### Drilling during IODP 398

IODP Expedition 398 took place from December 2022 to February 2023 aboard the research vessel *JOIDES Resolution*. The team drilled 12 sites in and around the Christiana–Santorini–Kolumbo volcanic field. Standard shipboard measurements of physical properties were carried out on the recovered cores, and core descriptions followed established pyroclastic classification. These descriptions accounted for common drilling disturbances and recovery artefacts such as sediment mixing, shear deformation, brecciation, biscuiting and ash liquefaction^48^.

To improve overall recovery of the caldera stratigraphy, multiple holes with small horizontal offsets (~50 m) were planned at each site. However, difficult drilling conditions meant that only a single hole was completed at sites U1594 and U1597, located inside the caldera. Site U1595 was drilled with three holes, and Site U1596 with two.

Stratigraphic correlation was used to align overlapping sections from adjacent holes and construct the most complete possible sedimentary record. This process relied mainly on shipboard physical property data and core images. Measurements were collected using the whole-round multisensor logger (WRMSL), which provided magnetic susceptibility and gamma-ray attenuation data, and a gamma-ray track system, which recorded natural gamma radiation.

Using these data, depth adjustments were applied as needed to develop a composite depth scale. This allowed the construction of a continuous stratigraphic section by splicing together the most representative intervals from multiple holes at each site.

The cores were sampled at specified locations or intervals, keeping as best as possible to a homogenous lithology, with a couple of grams of material taken per sample. Samples were taken with a standard 1cm-wide plastic 5 cm^3^ scoop, and all analyses were conducted on bulk sediment.

### Geochemistry

All sediment samples (*n* = 298, depth range = 0.25–126.43 mbsf) from Site U1595 (Holes A, B, C) were oven-dried at low temperatures (40 °C). Roughly 1 g of each bulk sample was ground to a fine powder by agate mortar and pestle, and 0.1 g used for each analysis in the labs at the University of Oxford. A slightly larger sampling focus was put onto Unit L3 since, due to lithology, L3 is more suitable for preserving hydrothermal signals compared to volcanic layers.

For Hg analyses, the LUMEX RA-915M with PYRO-915+ attachment Hg analyser was used. Mercury analysis followed the protocol^41^ where the sample is pyrolysed at 700 °C and Hg quantified using atomic absorption spectrometry. The instrument was calibrated with NIST SRM 2587 (paint-contaminated soil) with a reference concentration of 290 ± 9 ppb (ng/g). The percentage error for long-term observations of certified reference material NIST 2587, was ~5% and sample duplicates had a relative standard deviation of <±10%. Samples very close to the detection limit (<2 ppb), showed reproducibility of ~20% based on repeat measurements of the samples. Full Hg data in Source Data for Fig. 4.

Trace metal analysis was carried out on 40 samples in two rounds using a PerkinElmer NexION 350D Inductively Coupled Plasma Mass Spectrometer (ICP-MS) following a two-step heated acid digestion: 1:3 HF:HNO_3_ followed by 50% HNO_3_. Each sample was blank corrected to remove memory artefacts and paired with internal standards to normalise instrument drift. The metals analysed by the first round of ICP-MS were Fe, Mn, P, Sc, V, Co, Cu, Zn, As, Mo, Ag, Sb, Ba, Au, Pb. The elements Al, K, Na, Mg, Sr, Cd, Li and Ni were only analysed in the second round, so only half of the samples have measurements for these elements. Detection limits and standard deviations were calculated using the data obtained for the 39 down-hole samples by ICP-MS. In the first ICP-MS run, all elements analysed were above their respective detection limits. Percentage error for certified reference materials (BCR-2, SLRS-6) was ≤13.7% for all elements except for As, V and Zn. As was ±17.9%, however, its certified value was below the method quantification limit so a lower accuracy was expected. V and Zn had percentage errors compared to the certified value of ±20.6% and 27.1%, respectively for standard material SLRS-6, but percentage errors of only ±4.5% and 3.5% for BCR-2. The certified reference materials had a precision (relative standard deviation) of ≤4% for repeats of all elements. Sample duplicates (where the same sample was digested twice and run separately on the ICP-MS) and instrument duplicates (where the sample was measured twice by the machine) were also conducted. Sample duplicates were found to have a relative standard deviation of ≤6.5%, except for Pb in one sample duplicate that had a precision of ≤15.6%. Instrument duplicates were all ≤3.1%. Blanks were ≤2.5 ppb.

In the second ICP-MS run, all elements in the samples were above their respective detection limits except for Au, so Au was excluded from further analysis. Percentage error was found to be ≤12.7%, except for As, V, Zn and Cd, as well as some of the major elements for certified reference materials (BCR-2, SLRS-6). As and V had percentage errors of ±21.6% and 20.8%, respectively in SLRS-6, but both these elements were below the method quantification limit, so lower accuracy was expected; however, V had a percentage error of ±7% in BCR-2. Similarly, Zn had a percentage error of ±28.8% in SLRS-6 but only ±8.2% in BCR-2. This was a similar pattern to the first ICP-MS run. Cd was below the detection limit in SLRS-6, but had a percentage error of ±25.9% in BCR, and Mo went over range in BCR-2. For the major elements (Na, Mg, Al, K, Mn, Fe), they all had percentage errors ≤27.2% in BCR-2, but in SLRS-6, they showed much better accuracy, with an error of only ≤5.9%, highlighting the importance of having more than one type of standard when conducting analysis. The certified reference materials had a precision of ≤6.7% for repeats. Sample duplicates proved to be more variable due to the heterogeneous nature of the samples in the second run of ICP-MS. The highest precision was found to be ±11.42% for Sb. However, this was for a sample whose concentrations were very close to the detection limit and below the method quantification limit, and thus, lower precision was expected. Most other sample repeats were ≤10%. Instrument duplicates peaked at 14.5% for Mo, whose value was close to the method quantification limit. Most other duplicates were ≤10%. Blanks were ≤2.5 ppb. For both ICP-MS runs, only elements that showed a coefficient of variation over 0.3 were included in graphs and comparisons. For full ICP-MS data, including all accuracy and precision values, see Supplementary Data 1 and 2.

To better identify trace element anomalies, the values obtained were normalised against the 726 CE eruption (Unit L2) of the Kameni Islands, which represents the most recent sedimentation signature for the caldera. The Kameni Island products have been shown to have consistent compositions over the last ~2000 years^69^. Since Kameni products make up most of the ~130 m worth of caldera sediment-fill, the 726 CE was used as a ‘background’ value for Santorini’s intra-caldera sediments. Raw element concentrations are shown in Supplementary Fig. 1. The normalisation was carried out by averaging all 726 CE values for each element obtained from the ICP-MS run and dividing all other values for each element by this number. For the full 726 CE normalised dataset, see Source Data for Fig. 2.

Aluminium is usually used as a reference element, since it is largely inert and non-anthropogenic, when normalising concentration data to calculate an enrichment factor (denotes concentration):

2 $${Enrichment}\,{factor}\,{of}{element}\,X\,=\,\frac{({Sample}\,[X]/{Sample}\,\left[{Al}\right])}{({Background}\,[X]/{Background}[{Al}])}$$

However, this method could only be applied to 22 samples from the second ICP-MS run, and the resulting figure is shown in Supplementary Fig. 5. The patterns and enrichment factors are consistent with those in Fig. 2.

### Microbiology

A whole-round core sample from Unit L3 at 55 mbsf was chosen for metagenomic analysis from Hole U1595C and transferred immediately into a sterile Whirl-Pak bag and then into the hard-shell anaerobic chamber (COY anaerobic chamber rigid glove box with purge airlock, COY Laboratory Products Inc.) on board the R/V JOIDES Resolution. The outside of each core was sprayed three times with 70% ethanol. The core interior was carefully collected using an ethanol-sterilised stainless-steel spatula and immediately stored at −80 °C for shore-based DNA extraction^48^. Approximately 500 ng of high molecular weight DNA (>23 kb) was isolated from 50 g of sediment material^70^. Sequencing was performed using Illumina NovaSeq 6000 S4 PE150 XP platform, and 31,632,300 reads were produced. The assembly of the sequencing data and the annotation of the assembled sequences were performed using the IMG/M metagenome analysis system. A set of metagenomic libraries, previously constructed from samples collected during sampling expeditions of the IMBBC-HCMR in collaboration with the Joint Genome Institute, Department of Energy, USA (JGI, DoE), were also included in the analysis for comparison. These included: (a) the red mat sample RED, retrieved from the sea-bottom of the North Santorini caldera (36.4499° N, 25.4021° E, depth: 336 m); (b) the microbial mat sample V59, covering the surface of the sulphide chimney V59 in the active hydrothermal vent area of Kolumbo volcano (36.5263° N, 25.4867° E, depth: 505 m); (c) the water sample VENT, collected from the water column, at an altitude of 30 m above the active vent chimneys of Kolumbo (36.5264° N, 25.4868° E, depth: 470 m); and (d) the water sample W25, collected from the bottom of the inactive area of Kolumbo, at the southern part of its crater (36.527° N, 25.4871° E, depth: 495 m). Samples RED and V59 were selected as representatives of a high hydrothermal environment, the Vent sample as a distal vent water sample and sample W25 as a representative of a non-hydrothermal environment. All metagenomes were screened for a series of genes related to the *mer* system with KEGG Orthologs (KO) identifiers K07721, K00221, K19059, K19057, K19058, K08363, K00520 and K008365, to arsenate As(V) reductase with K00537 and K03741, to heavy metal efflux systems for Co, Zn, Cd with K15726, K16234, K15725 and K15727, to Cu resistance and efflux with pfam13743, K17686, K07787 and K07798, to Mo co-factor associated metabolism with K03750, to Fe resistance (Fe reduction and extracellular electron transfer) with K18915, pfam13435, pfam11854, pfam14522, K04758 and K04759 and to Mn transport with K03322. Taxonomic assignment of the *mer*A, *ars*C, *czc* complex, Cu-binding, *cop*A, *cus*A, *cus*B, *moe*A, *fer*R, *mtr*A, *mtr*B, *mtr*C, *feo*A, *feo*B and *mnt*H genes of the deep subsurface sample U1595-Unit L3, available in the IMG/M system, were also retrieved at the domain, phylum, class, order, family, genus and species levels. All gene counts were normalised using a set of ribosomal protein reference genes with COG IDs: COG0048, COG0049, COG0051, COG0080, COG0089, COG0091, COG0093, COG0096, COG0098 and COG0099 for each metagenome. The reference datasets used in the present study are available in the IMG/M system with accessions 3300066567, 3300002231, 3300009702, 3300024344, 3300009703 for samples RED, V59, VENT and W25, respectively. For the data used to make Fig. 3, see Source Data for Fig. 3.

### Metal Budgets and Fluxes

The dry bulk density for representative samples from each core was analysed shipboard^48^. Within Unit L3, there were two densities provided—one in ash and one in tuffaceous mud. The tuffaceous mud density value was used in the calculations using Eq. 1 since it best represented our hydrothermal layers. For the post-726 CE values (Unit L1), the average of all the dry densities provided for the tuffaceous mud layers within the top 20 mbsf was taken and used in Eq. 1. The element anomaly over background was calculated by subtracting the average 726 CE Hg value (0.00075 ppm) from the average Hg value for the tuffaceous mud layers in each phase, the whole of Unit L3 and the post-726 CE values (Unit L1). This was repeated for the elements in Table 3 for Unit L3 and Unit L1.

For the comparison of Mn and Fe fluxes with previous work^45^ we used only core material available from the southern basin and recalculated values from published bulk sediment concentrations to avoid any sediment density or basin area differences. The only core material directly comparable to our newly generated data is from ‘Core 15’. Assuming a tuffaceous mud sedimentation rate of 0.122 cm yr^−1^, the first 45 cm (9 samples) represents ~370 years. We hence calculated the average Mn and Fe concentration values for the top 45 cm of that core, subtracted the quoted background value (taken outside the caldera) from the averages and assumed a dry bulk density equivalent to the top of the IODP core material (700 kg m^3^) rather than a generic wet density value. This yielded fluxes for Fe from 553 to 88.9 t yr^−1^ and for Mn from 35 to 3.85 t yr^−1^, respectively for the southern basin.

Five values were reported for Hg sediment accumulation rates in the Ionian Sea^68^, we excluded the final value from 1884 CE as it is in the midst of the industrial revolution and would skew the average. The Hg sediment accumulation values included to create the average of 1.1 µg m^−2^ yr^−1^ were from the years 945 CE (0.4 µg m^−2^ yr^−1^), 1179 CE (0.43 µg m^−2^ yr^−1^), 1414 CE (0.78 µg m^−2^ yr^−1^) and 1649 CE (2.8 µg m^−2^ yr^−1^). The average value was then multiplied by the size of the southern basin and entire caldera to obtain the estimated background Hg fluxes for Santorini. The calculations and data in Table 2 can be found in Source Data for Table 2.

### Reporting summary

Further information on research design is available in the Nature Portfolio Reporting Summary linked to this article.

## Supplementary information


Supplementary Information
Description of Additional Supplementary Files
Supplementary Data 1
Supplementary Data 2
Reporting Summary
Transparent Peer Review file


## Source data


Source Data


## Data Availability

The deep subsurface sequence reads of the present study (U1595C Unit L3) have been deposited to the IMG/M system with accession 3300066567 (https://img.jgi.doe.gov/cgi-bin/m/main.cgi?section=TaxonDetail&page=taxonDetail&taxon_oid=3300066567) and to the National Centre for Biotechnology Information Sequence Read Archive under access numbers SRR37198718, Biosample SAMN55199529 (https://www.ncbi.nlm.nih.gov/biosample/55199529) and Bioproject PRJNA1419865 (https://www.ncbi.nlm.nih.gov/bioproject/1419865). The reference datasets used in the present study are available in the IMG/M system with accessions 3300002231, 3300009702, 3300024344, 3300009703 for samples RED, V59, VENT and W25, respectively. Source data are provided with this paper.

## References

[1] de Ronde, C. E. J. & Stucker, V. K. Seafloor hydrothermal venting at volcanic arcs and backarcs. in *The Encyclopedia of Volcanoes* 2nd edn (ed. Sigurdsson, H.) Ch. 47, 823–849. 10.1016/B978-0-12-385938-9.00047-X (Academic Press, 2015).

[2] Caramanna, G., Sievert, S. M. & Bühring, S. I. Submarine shallow-water fluid emissions and their geomicrobiological imprint: a global overview. *Front. Mar. Sci*. **8**, 727199 (2021).

[3] German, C. R. & Seyfried, W. E. 8.7 - Hydrothermal Processes. in *Treatise on Geochemistry* 2nd edn (eds Holland, H. D. & Turekian, K. K.) 191–233. 10.1016/B978-0-08-095975-7.00607-0 (Elsevier, 2014).

[4] Farquharson, J. I., Heap, M. J., Lavallée, Y., Varley, N. R. & Baud, P. Evidence for the development of permeability anisotropy in lava domes and volcanic conduits. *J. Volcanol. Geotherm. Res.* **323**, 163–185 (2016).

[5] Walter, T. R. et al. Complex hazard cascade culminating in the Anak Krakatau sector collapse. *Nat. Commun.* **10**, 4339 (2019). 31575866 10.1038/s41467-019-12284-5 PMC6773710

[6] Beaulieu, S. E., Baker, E. T., German, C. R. & Maffei, A. An authoritative global database for active submarine hydrothermal vent fields. *Geochem. Geophys. Geosyst.* **14**, 4892–4905 (2013).

[7] Smith, P. A. & Cronan, D. S. The geochemistry of metalliferous sediments and waters associated with shallow submarine hydrothermal activity (Santorini, Aegean Sea). *Chem. Geol.* **39**, 241–262 (1983).

[8] Cronan, D. S., Varnavas, S. P. & Hodkinson, R. Hydrothermal mineralizing processes and associated sedimentation in the santorini hydrothermal embayments. *Mar. Georesources Geotechnol.* **18**, 77–118 (2000).

[9] Nomikou, P. et al. SANTORY: SANTORini’s seafloor volcanic observatory. *Front. Mar. Sci.* **9**, 796376 (2022).

[10] Simmons, S. F. & Brown, K. L. The flux of gold and related metals through a volcanic arc, Taupo Volcanic Zone, New Zealand. *Geol* **35**, 1099 (2007).

[11] Cronan, D. S., Johnson, A. G. & Hodkinson, R. A. Hydrothermal fluids may offer clues about impending volcanic eruptions. *Eos Trans. Am. Geophys. Union* **78**, 341–345 (1997).

[12] Henley, R. W. et al. The 15 January 2022 Hunga (Tonga) eruption: a gas-driven climactic explosion. *J. Volcanol. Geotherm. Res.* **451**, 108077 (2024).

[13] Astorch-Cardona, A., Guerre, M., Dolla, A., Chavagnac, V. & Rommevaux, C. Spatial comparison and temporal evolution of two marine iron-rich microbial mats from the Lucky Strike Hydrothermal Field, related to environmental variations. *Front. Mar. Sci.* **10**, 1038192 (2023).

[14] Agusto, M. et al. Eleven-year survey of the magmatic-hydrothermal fluids from peteroa volcano: identifying precursory signals of the 2018–2019 eruption. *Geochem., Geophys. Geosyst.* **24**, e2023GC011064 (2023).

[15] Stucker, V. K., de Ronde, C. E. J., Laurence, K. J. & Phillips, A. M. Rare time series of hydrothermal fluids for a submarine volcano: 14 years of vent fluid compositions for brothers Volcano, Kermadec Arc, New Zealand. *Econ. Geol.* **118**, 1563–1576 (2023).

[16] de Ronde, C. E. J. et al. Evolution of a submarine magmatic-hydrothermal system: brothers volcano, southern Kermadec Arc, New Zealand. *Econ. Geol.* **100**, 1097–1133 (2005).

[17] de Ronde, C. E. J., Humphris, S. E., Höfig, T. W. & Reyes, A. G. & the IODP Expedition 376 Scientists. Critical role of caldera collapse in the formation of seafloor mineralization: the case of Brothers volcano. *Geology* **47**, 762–766 (2019).

[18] Hawkes, J. A., Connelly, D. P., Rijkenberg, M. J. A. & Achterberg, E. P. The importance of shallow hydrothermal island arc systems in ocean biogeochemistry. *Geophys. Res. Lett.* **41**, 942–947 (2014).

[19] Cronan, D. S., Varnavas, S. & Perissoratis, C. Hydrothermal sedimentation in the caldera of Santorini, Hellenic Volcanic Arc. *Terra Nova* **7**, 289–293 (1995).

[20] Hammer, C. U., Clausen, H. B., Friedrich, W. L. & Tauber, H. The Minoan eruption of Santorini in Greece dated to 1645 BC?. *Nature* **328**, 517–519 (1987).

[21] Druitt, T. H. et al. Chapter 2 Geological and tectonic setting of Santorini. *Geol. Soc. Lond. Mem.* **19**, 5–12 (1999).

[22] Druitt, T. & Francaviglia, V. Caldera formation on Santorini and the physiography of the islands in the late Bronze Age. *Bull. Volcano.* **54**, 484–493 (1992).

[23] Hooft, E. E. E. et al. Seismic imaging of Santorini: subsurface constraints on caldera collapse and present-day magma recharge. *Earth Planet. Sci. Lett.* **514**, 48–61 (2019).

[24] Druitt, T. H. New insights into the initiation and venting of the Bronze-Age eruption of Santorini (Greece), from component analysis. *Bull. Volcano.* **76**, 794 (2014).

[25] Karátson, D. et al. Towards reconstruction of the lost Late Bronze Age intra-caldera island of Santorini, Greece. *Sci. Rep.* **8**, 7026 (2018). 29728639 10.1038/s41598-018-25301-2 PMC5935677

[26] Varnavas, S. P. & Cronan, D. S. Submarine hydrothermal activity off Santorini and Milos in the Central Hellenic Volcanic Arc: a synthesis. *Chem. Geol.* **224**, 40–54 (2005).

[27] Nomikou, P. et al. The emergence and growth of a submarine volcano: the Kameni islands, Santorini (Greece). *GeoResJ* **1–2**, 8–18 (2014).

[28] Washington, H. S. Santorini eruption of 1925. *Geol. Soc. Am. Bull.* **37**, 349–384 (1926).

[29] Varnavas, S. P. & Cronan, D. S. Arsenic, antimony and bismuth in sediments and waters from the Santorini hydrothermal field, Greece. *Chem. Geol.* **67**, 295–305 (1988).

[30] Barkay, T., Miller, S. M. & Summers, A. O. Bacterial mercury resistance from atoms to ecosystems. *FEMS Microbiol. Rev.* **27**, 355–384 (2003). 12829275 10.1016/S0168-6445(03)00046-9

[31] Schroll, E. Contribution to the mineralogy of the ron-rich mud sediments of Santorini, Greece. *Thera Aegean World* **1**, 333–342 (1978).

[32] Sigurdsson, H. et al. Marine investigations of Greece’s Santorini Volcanic Field. *Eos Trans. Am. Geophys. Union* **87**, 337–342 (2006).

[33] Camilli, R. et al. The Kallisti Limnes, carbon dioxide-accumulating subsea pools. *Sci. Rep.* **5**, 12152 (2015). 26179858 10.1038/srep12152 PMC4503996

[34] Roberts, H., Price, R., Brombach, C.-C. & Pichler, T. Mercury in the hydrothermal fluids and gases in Paleochori Bay, Milos, Greece. *Mar. Chem.* **233**, 103984 (2021).

[35] Cox, M. E. & McMurtry, G. M. Vertical distribution of mercury in sediments from the East Pacific Rise. *Nature* **289**, 789–792 (1981).

[36] Gamboa Ruiz, W. L. & Tomiyasu, T. Distribution of mercury in sediments from Kagoshima Bay, Japan, and its relationship with physical and chemical factors. *Environ. Earth Sci.* **74**, 1175–1188 (2015).

[37] Lim, D., Kim, H., Kim, J., Jeong, D. & Kim, D. Mercury proxy for hydrothermal and submarine volcanic activities in the sediment cores of Central Indian Ridge. *Mar. Pollut. Bull.* **159**, 111513 (2020). 32777546 10.1016/j.marpolbul.2020.111513

[38] Kotnik, J. et al. Mercury speciation in surface and deep waters of the Mediterranean Sea. *Mar. Chem.* **107**, 13–30 (2007).

[39] Stoffers, P. et al. Elemental mercury at submarine hydrothermal vents in the Bay of Plenty, Taupo volcanic zone, New Zealand. *Geol* **27**, 931 (1999).

[40] Outridge, P. M. & Wang, F. The stability of metal profiles in freshwater and marine sediments. in *Environmental Contaminants* Vol. 18 (eds Blais, J. M., Rosen, M. R. & Smol, J. P.) 35–60 (Springer Netherlands, 2015).

[41] Frieling, J., Fendley, I. M., Nawaz, M. A. & Mather, T. A. Assessment of Hg speciation changes in the sedimentary rock record from thermal desorption characteristics. *Geochem. Geophys. Geosyst.* **25**, e2024GC011502 (2024).

[42] Torres-Rodriguez, N. et al. Natural iron fertilization moderates hydrothermal mercury inputs from arc volcanoes. *Environ. Sci. Technol.* **59**, 11039–11050 (2025). 40437961 10.1021/acs.est.5c01767

[43] Torres-Rodriguez, N. et al. Mercury fluxes from hydrothermal venting at mid-ocean ridges constrained by measurements. *Nat. Geosci*. 1–7. 10.1038/s41561-023-01341-w (2023).

[44] Arvanitides, N. et al. Geochemistry of Lavas, Pumice and Veins in Drill Core GPK-1, Palaea Kameni, Santorini. In *Thera and the Aegean World III* (ed. by Hardy, D. A.) Vol. 2, 266–279 (The Thera Foundation, London, 1990).

[45] Petersen, M. D. & Muller, G. Recent Tuffitic Sediments around Santorini, Greece. Part IV. Geochemistry of iron-rich sediments from the Santorini Caldera. In *Thera and the Aegean World I* (ed. by C. Doumas) 311–332 (The Thera Foundation, London, 1978).

[46] Hanert, H. H. Bacterial and chemical iron oxide deposition in a shallow bay on Palaea Kameni, Santorini, Greece: microscopy, electron probe microanalysis, and photometry of in situ experiments. *Geomicrobiol. J.* **19**, 317–342 (2002).

[47] Robbins, E. I. et al. From Precambrian iron-formation to terraforming Mars: the JIMES expedition to Santorini. *Geomicrobiol. J.* **33**, 1–16 (2016).

[48] Druitt, T. H., Kutterolf, S., Ronge, T. A., & Expedition 398 Scientists. *Hellenic Arc Volcanic Field* Vol. 398 (International Ocean Discovery Program, 2024).

[49] Preine, J. et al. Hazardous explosive eruptions of a recharging multi-cyclic island arc caldera. *Nat. Geosci*. 1–9. 10.1038/s41561-024-01392-7 (2024).

[50] Nomikou, P. et al. Post-eruptive flooding of Santorini caldera and implications for tsunami generation. *Nat. Commun.* **7**, 13332 (2016). 27824353 10.1038/ncomms13332 PMC5105177

[51] Hector, S. et al. Unravel subseafloor hydrothermal leaching and magmatic degassing during chimney formation at Kolumbo volcano. *Sci. Rep.* **15**, 14673 (2025). 40287518 10.1038/s41598-025-99586-5 PMC12033240

[52] Deng, C. et al. Recycling of mercury from the atmosphere-ocean system into volcanic-arc–associated epithermal gold systems. *Geology* **49**, 309–313 (2020).

[53] Henley, R. W. & Berger, B. R. Nature’s refineries—metals and metalloids in arc volcanoes. *Earth-Sci. Rev.* **125**, 146–170 (2013).

[54] Hodkinson, R. A., Cronan, D. S., Varnavas, S. & Perissoratis, C. Regional geochemistry of sediments from the Hellenic volcanic arc in regard to submarine hydrothermal activity. *Mar. Georesources Geotechnol.* **12**, 83–129 (1994).

[55] Boyd, E. S. & Barkay, T. The mercury resistance operon: from an origin in a geothermal environment to an efficient detoxification machine. *Front. Microbiol.* **3**, 349 (2012). 23087676 10.3389/fmicb.2012.00349 PMC3466566

[56] Sevak, P. & Pushkar, B. Bacterial responses towards arsenic toxicity and in-depth analysis of molecular mechanism along with possible on-field application. *J. Environ. Chem. Eng.* **11**, 110187 (2023).

[57] Besaury, L. et al. Abundance and diversity of copper resistance genes *cus*A and *cop*A in microbial communities in relation to the impact of copper on Chilean marine sediments. *Mar. Pollut. Bull.* **67**, 16–25 (2013). 23298430 10.1016/j.marpolbul.2012.12.007

[58] Nies, D. H. The cobalt, zinc, and cadmium efflux system CzcABC from Alcaligenes eutrophus functions as a cation-proton antiporter in Escherichia coli. *J. Bacteriol.* **177**, 2707–2712 (1995). 7751279 10.1128/jb.177.10.2707-2712.1995 PMC176940

[59] Sestok, A. E., Linkous, R. O. & Smith, A. T. Toward a mechanistic understanding of Feo-mediated ferrous iron uptake. *Metallomics* **10**, 887–898 (2018). 29953152 10.1039/c8mt00097b PMC6051883

[60] Ross, D. E. et al. Characterization of protein-protein interactions involved in iron reduction by Shewanella oneidensis MR-1. *Appl. Environ. Microbiol.* **73**, 5797–5808 (2007). 17675441 10.1128/AEM.00146-07 PMC2074908

[61] Hohle, T. H. & O’Brian, M. R. The mntH gene encodes the major Mn2+ transporter in Bradyrhizobium japonicum and is regulated by manganese via the Fur protein. *Mol. Microbiol.* **72**, 399–409 (2009). 19298371 10.1111/j.1365-2958.2009.06650.x PMC2675660

[62] Nichols, J. & Rajagopalan, K. V. Escherichia coli MoeA and MogA. Function in metal incorporation step of molybdenum cofactor biosynthesis. *J. Biol. Chem.* **277**, 24995–25000 (2002). 12006571 10.1074/jbc.M203238200

[63] Dutrieux, A. M. et al. Metal preservation and mobilization in sediments at the TAG hydrothermal field, Mid-Atlantic Ridge. *Geochem. Geophys. Geosyst.* **24**, e2023GC010879 (2023).

[64] Polymenakou, P. N. et al. Taxonomic diversity of microbial communities in sub-seafloor hydrothermal sediments of the active Santorini-Kolumbo volcanic field. *Front. Microbiol*. **14**, 1188544 (2023). 10.3389/fmicb.2023.1188544 PMC10345502 37455712

[65] Rohling, E. J., Marino, G., Grant, K. M., Mayewski, P. A. & Weninger, B. A model for archaeologically relevant Holocene climate impacts in the Aegean-Levantine region (easternmost Mediterranean). *Quat. Sci. Rev.* **208**, 38–53 (2019).

[66] Koschinsky, A., Schmidt, K. & Garbe-Schönberg, D. Geochemical time series of hydrothermal fluids from the slow-spreading Mid-Atlantic Ridge: implications of medium-term stability. *Chem. Geol.* **552**, 119760 (2020).

[67] Tomiyasu, T., Mitsui, A., Mitarai, M., Kodamatani, H. & Kanzaki, R. Seasonal variation in mercury species in seawater of Kagoshima Bay, southern Kyushu, Japan: the impact of active submarine volcanos on the inner bay. *Mar. Chem.* **244**, 104133 (2022).

[68] Cossa, D. et al. Mercury deposition in the Eastern Mediterranean: modern fluxes in the water column and Holocene accumulation rates in abyssal sediment. *Chem. Geol.* **636**, 121652 (2023).

[69] Barton, M. & Huijsmans, J. P. P. Post-caldera dacites from the Santorini volcanic complex, Aegean Sea, Greece: an example of the eruption of lavas of near-constant composition over a 2,200 year period. *Contrib. Mineral. Petrol.* **94**, 472–495 (1986).

[70] Brady, S. F. Construction of soil environmental DNA cosmid libraries and screening for clones that produce biologically active small molecules. *Nat. Protoc.* **2**, 1297–1305 (2007). 17546026 10.1038/nprot.2007.195

