# Supplementary material for: A prolonged hydrothermal past at Santorini Caldera revealed by sedimentary trace metal and microbial signatures
Source: Nat Commun. 2026 Aug 4;17:9398. doi: 10.1038/s41467-026-75931-8 (PMC13534618; doi:10.1038/s41467-026-75931-8)
Supplement: Supplementary file 1 [file 41467_2026_75931_MOESM1_ESM.pdf]

A prolonged hydrothermal past at Santorini Caldera revealed by sedimentary trace metal and microbial signatures

Della Sala et al.

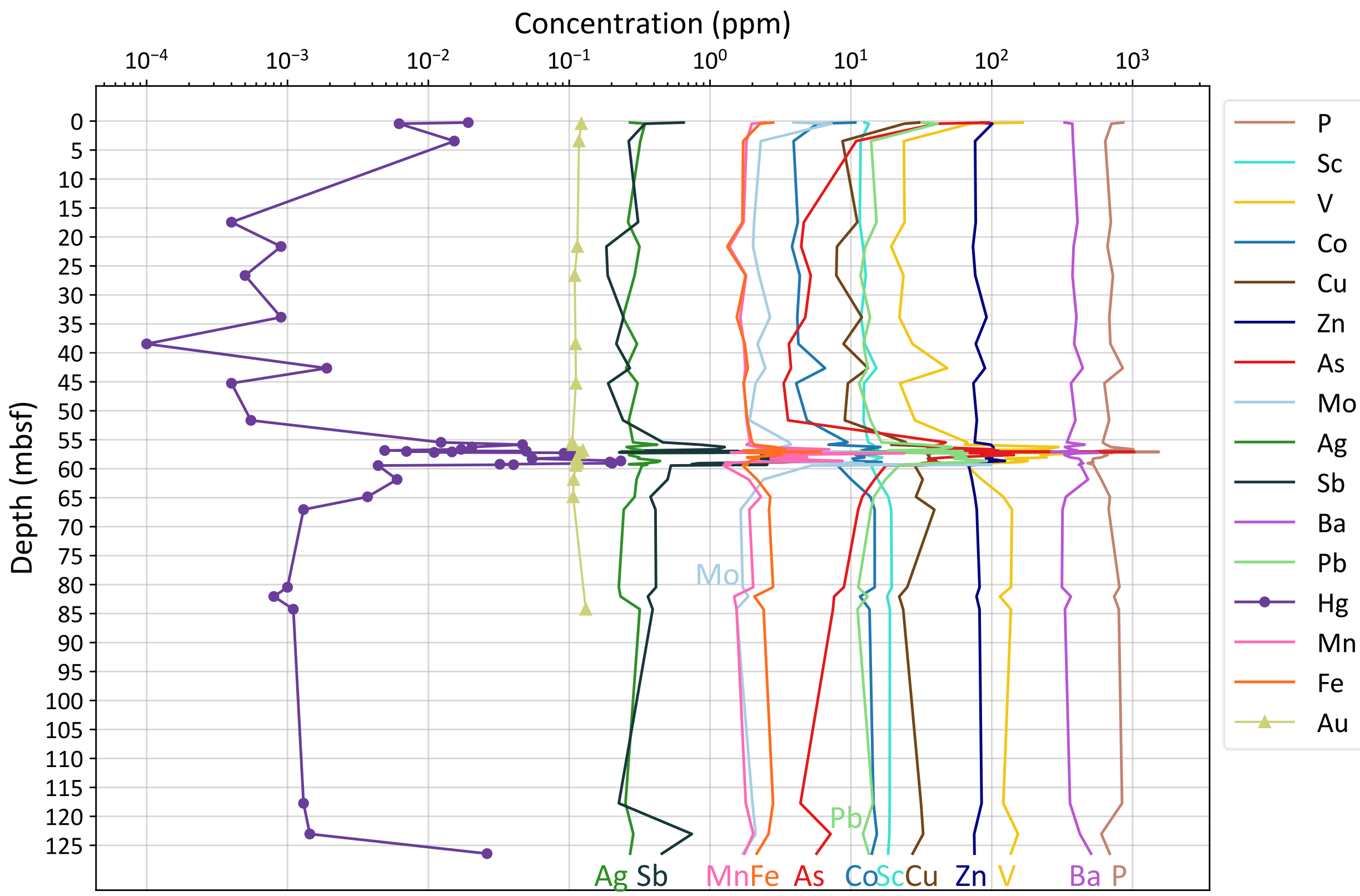

Supplementary Fig. 1: **Raw element concentrations of site U1595**. 0–90 mbsf from Hole U1595A, 108–127 mbsf from Hole U1595B. Key provided, highlighting which colour corresponds to which element. Elements labelled directly on the graph as well for easy identification. Au values are only from the first run of ICP-MS since it was below the detection limit in the second run. Concentration along the x-axis is displayed as a log scale, and concentrations are given in ppm

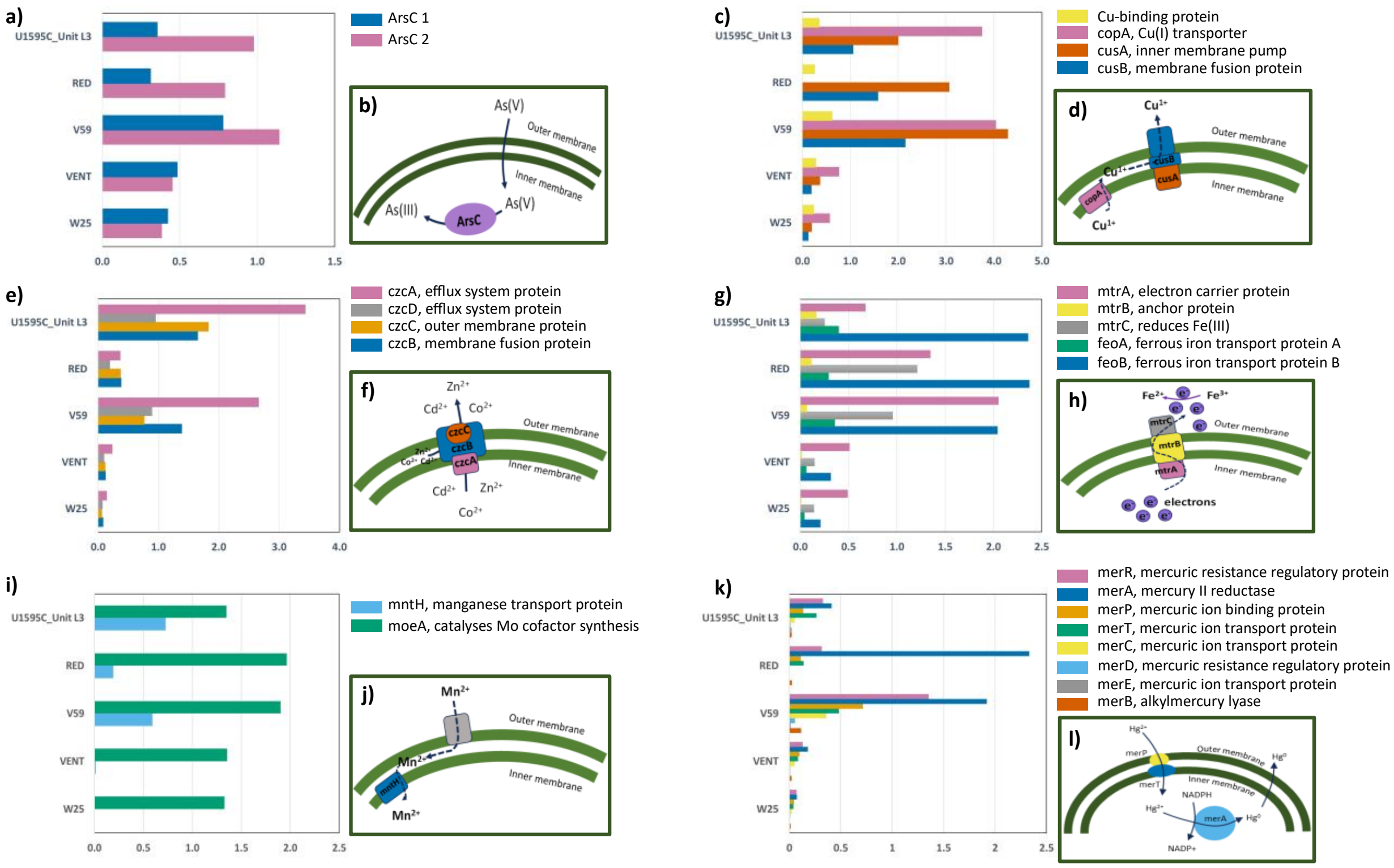

Supplementary Fig. 2. Metal resistance and metal redox-related genes. Normalised gene counts of metal resistance and metal-associated genes in the Unit L3 sample from Hole U1595C and the reference samples RED, V59, VENT and W25 for **a)** arsenic detoxification (arsenate As(V) reductase: *ars*C variants), **c)** copper resistance and efflux systems (copA, *cus*A-B and cupredoxin-like proteins), **e)** cobalt–zinc–cadmium efflux systems (*czc*A-C), **g)** iron reduction and extracellular electron transfer (*mtr*A-C, *feo*A-B), **i)** manganese transport (*mnt*H), and molybdenum cofactor-associated metabolism (*meo*A), and **k)** mercury detoxification system (*mer*A-E and *mer*T,P,R). Schematic representations of the corresponding metal resistance or metal redox pathways for **b)** arsenate detoxification, **d)** copper efflux systems, **f)** cobalt, zinc, cadmium efflux system, **h)** extracellular Fe(III) reduction via the *mtr* complex, **j)** manganese inner transport and **l)** mercury detoxification.

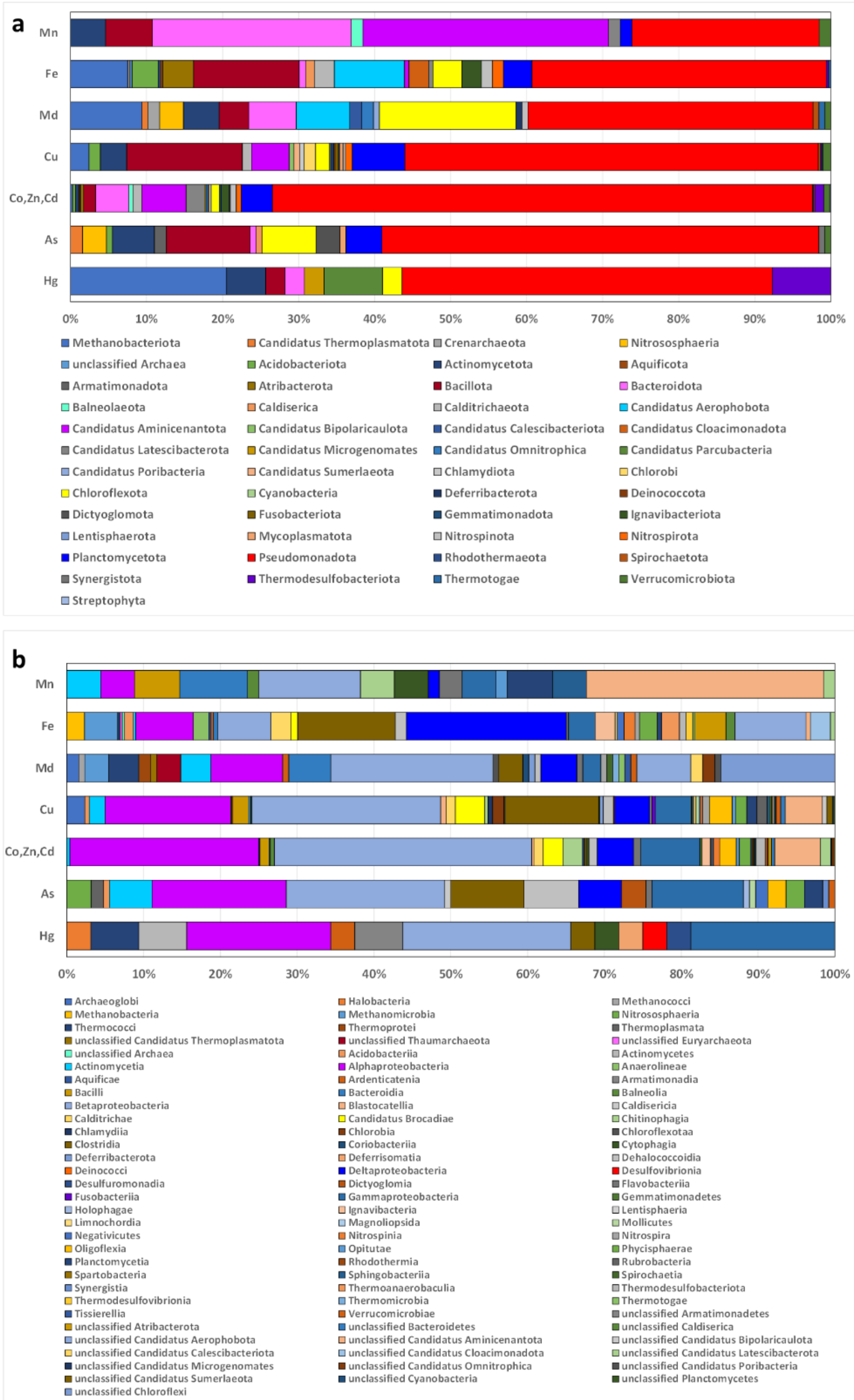

Supplementary Fig. 3: Taxonomic assignment of metal resistance genes. Relative abundances of taxonomically assigned metal resistance and metal-related genes (Mn, Fe, Mo, Cu, Co-Zn-Cd, As, Hg) retrieved from the deep subsurface metagenome. **a)** at the phylum level and **b)** at the class level, providing higher taxonomic resolution of the dominant microbial groups. Taxonomic assignments were obtained using the IMG/M metagenome data management system and correspond to best available matches based on sequence similarity. They do not imply exact nucleotide or amino acid identity to cultured representatives or species-level resolution. Source Data can be found in Source Data for Supplementary Fig. 3.

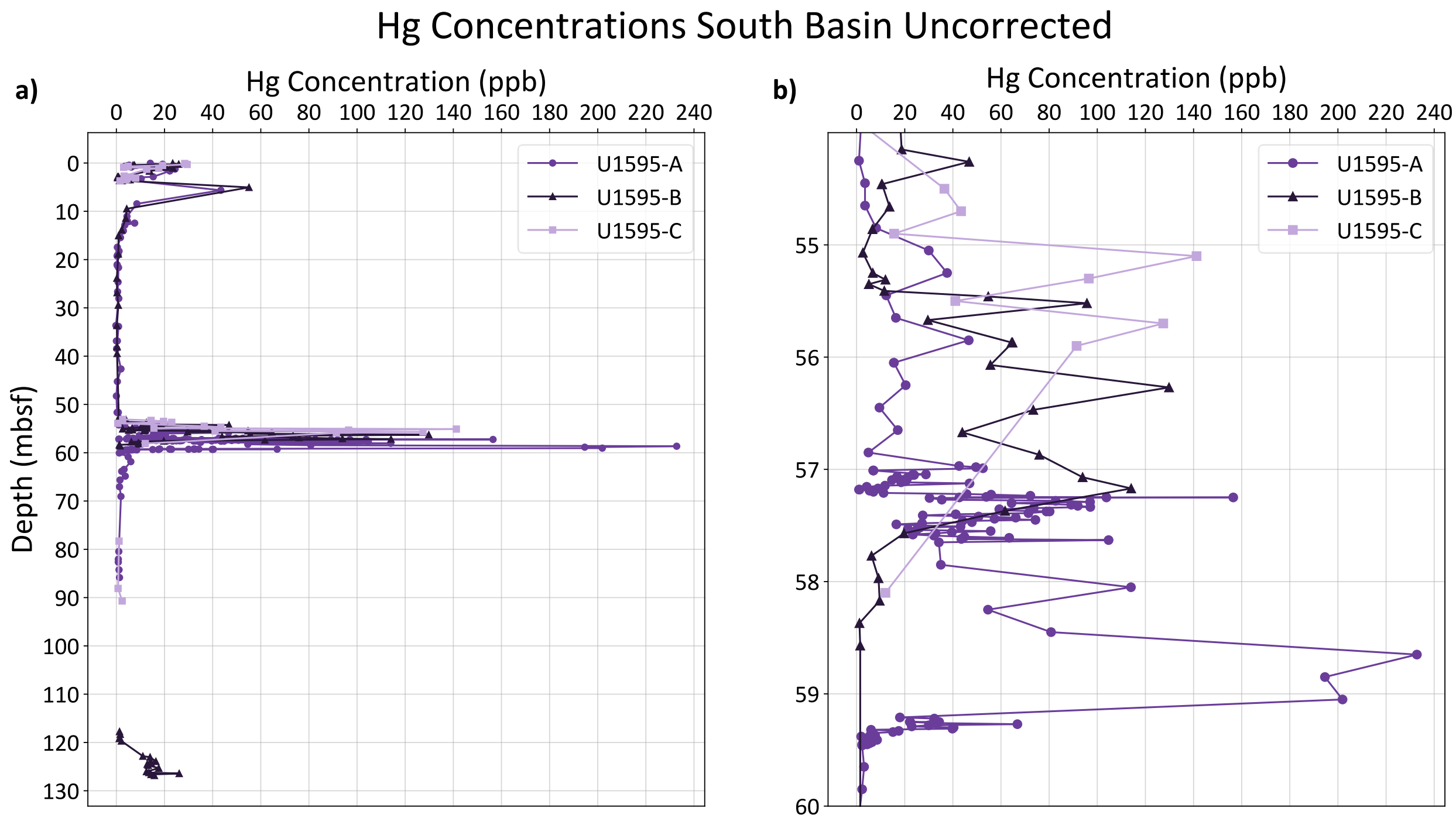

Supplementary Fig. 4: Uncorrected plots for Hg at site U1595. a) The whole sequence showing the full depth of the site and the Hg concentrations for each hole: U1595A, U1595B, U1595C. Breaks in the line indicate sampling gaps and/or that section of core was not recovered. b) A zoomed-in section covering from depth 55 to 60mbsf, to be able to see in detail large Hg peak at those depths in all the holes at that site. All depths are original depths.

Supplementary Table 1: Timescales of each hydrothermal phase. Time in years of hydrothermal pulses and periods of quiescence (interphases) in between correlated to size of tuffaceous mud layer. Timescale is calculated by using the 0.122 cm/year sediment accumulation rate, and is rounded to the nearest 10.

Phase	Tuffaceous mud (cm)	Timescale (years)
1	3	30
Interphase	2	20
2a	45	370
Interphase	19	160
2b	37	300
Interphase	15	120
2c	2	20

Interphase	10	80
2d	15	120
Interphase	4.5	40
3a	3	20
Interphase	7.5	60
3b	2	20

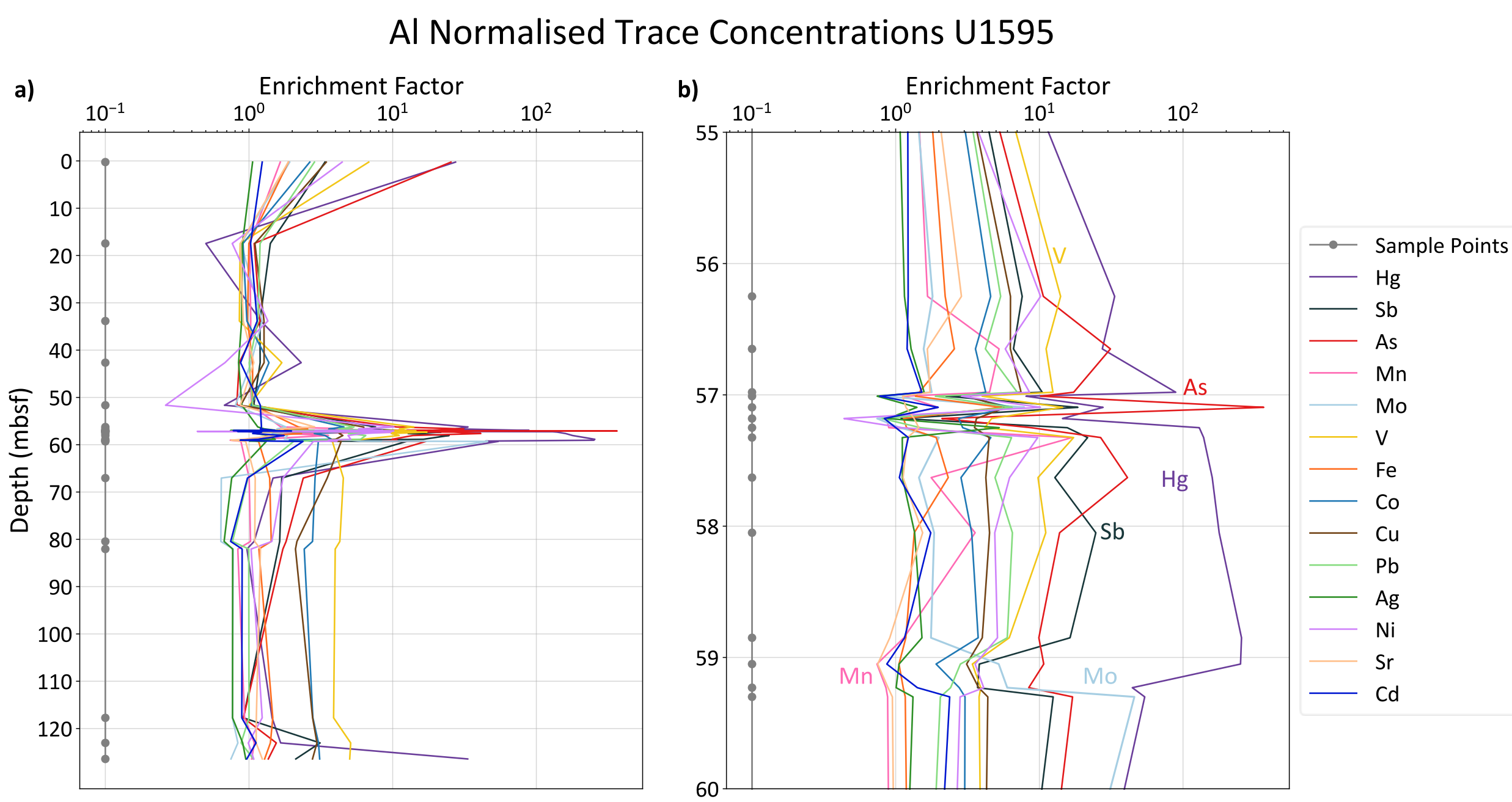

Supplementary Fig. 5: 726 CE-normalised trace metals at Site U1595 using reference element Al. 0–90 mbsf from Hole U1595A, 108–127 mbsf from Hole U1595B. Depth of sampling denoted by the grey line labelled 'Sample Points'. **a)** The element enrichment factors down-core. **b)** The elements in a zoomed-in interval spanning Unit L3 (55-60 mbsf). The x-axis on both graphs is a log scale. A key is provided and elements labelled on the graph. The elements provided are the same as those in Fig. 2, along with some elements with a coefficient of variation over 0.3 that were only measured in the second ICP-MS run.
