# Supplementary material for: A prolonged hydrothermal past at Santorini Caldera revealed by sedimentary trace metal and microbial signatures
Source: Nat Commun. 2026 Aug 4;17:9398. doi: 10.1038/s41467-026-75931-8 (PMC13534618; doi:10.1038/s41467-026-75931-8)
Supplement: Supplementary file 2 [file 41467_2026_75931_MOESM2_ESM.pdf]

Description of Additional Supplementary Files:

Supplementary Data 1: The raw concentrations for trace elements from the two ICP-MS runs (shown on separate sheets in this workbook). n= 17 with two sample duplicates and two intrument duplictaes for ICP-MS Run 1, and n=25 for ICP-MS Run 2. Two samples from Run 1 were repeated in Run 2 so 40 unique samples. Section headings for the data on the left-hand side in column A. The concentration units in bold by the section heading are the ones relevant to that section. The different duplicates are defined in the methods of the main text. SLRS-6 is a standard introduced directly into the machine, whilst BCR-2 was digested as a sample. Quality Control is another standard reference material used to ensure accuracy throughout the analysis. Memory/wash is the instrument background that is used to calculate the detection and quantification limits of the analysis. AVG = average, STD = standard deviation, %RSD = percentage relative standard deviation.

Supplementary Data 2: The raw concentrations for major elements from the two ICP-MS runs (shown on separate sheets in this workbook). n= 17 with two sample duplicates and two intrument duplictaes for ICP-MS Run 1, and n=25 for ICP-MS Run 2. Two samples from Run 1 were repeated in Run 2 so 40 unique samples. Section headings for the data on the left-hand side in column A. The concentrations in bold by the section heading are the ones relevant to that section. The different duplicates are defined in the methods of the main text. SLRS-6 is a standard introduced directly into the machine, whilst BCR-2 was digested as a sample. Quality Control is another standard reference material used to ensure accuracy throughout the analysis. Memory/wash is the instrument background that is used to calculate the detection and quantification limits of the analysis. AVG = average, STD = standard deviation, %RSD = percentage relative standard deviation.
