# Supplementary material for: A prolonged hydrothermal past at Santorini Caldera revealed by sedimentary trace metal and microbial signatures
Source: Nat Commun. 2026 Aug 4;17:9398. doi: 10.1038/s41467-026-75931-8 (PMC13534618; doi:10.1038/s41467-026-75931-8)
Supplement: Supplementary file 3 [file 41467_2026_75931_MOESM5_ESM.pdf]

nature portfolio

Corresponding author(s): Sofia Della Sala

Last updated by author(s): Apr 24, 2026

Reporting Summary

Nature Portfolio wishes to improve the reproducibility of the work that we publish. This form provides structure for consistency and transparency in reporting. For further information on Nature Portfolio policies, see our Editorial Policies and the Editorial Policy Checklist.

Statistics

For all statistical analyses, confirm that the following items are present in the figure legend, table legend, main text, or Methods section.

n/a	Confirmed	
☐	☒	The exact sample size (*n*) for each experimental group/condition, given as a discrete number and unit of measurement
☐	☒	A statement on whether measurements were taken from distinct samples or whether the same sample was measured repeatedly
☒	☐	The statistical test(s) used AND whether they are one- or two-sided<br>*Only common tests should be described solely by name; describe more complex techniques in the Methods section.*
☐	☒	A description of all covariates tested
☐	☒	A description of any assumptions or corrections, such as tests of normality and adjustment for multiple comparisons
☐	☒	A full description of the statistical parameters including central tendency (e.g. means) or other basic estimates (e.g. regression coefficient) AND variation (e.g. standard deviation) or associated estimates of uncertainty (e.g. confidence intervals)
☒	☐	For null hypothesis testing, the test statistic (e.g. *F*, *t*, *r*) with confidence intervals, effect sizes, degrees of freedom and *P* value noted<br>*Give P values as exact values whenever suitable.*
☒	☐	For Bayesian analysis, information on the choice of priors and Markov chain Monte Carlo settings
☒	☐	For hierarchical and complex designs, identification of the appropriate level for tests and full reporting of outcomes
☒	☐	Estimates of effect sizes (e.g. Cohen's *d*, Pearson's *r*), indicating how they were calculated

Our web collection on statistics for biologists contains articles on many of the points above.

Software and code

Policy information about availability of computer code

Data collection	No software was used
Data analysis	No software was used

For manuscripts utilizing custom algorithms or software that are central to the research but not yet described in published literature, software must be made available to editors and reviewers. We strongly encourage code deposition in a community repository (e.g. GitHub). See the Nature Portfolio guidelines for submitting code & software for further information.

Data

Policy information about availability of data

All manuscripts must include a data availability statement. This statement should provide the following information, where applicable:

- Accession codes, unique identifiers, or web links for publicly available datasets
- A description of any restrictions on data availability
- For clinical datasets or third party data, please ensure that the statement adheres to our policy

The deep subsurface sequence reads of the present study (U1595C Unit
L3) have been deposited to the IMG/M system with accession 3300066567 (https://img.jgi.doe.gov/cgi-bin/m/main.cgi?section=TaxonDetail&page=taxonDetail&taxon_oid=3300066567)
and to the National Centre for Biotechnology Information Sequence Read

Archive under access numbers SRR37198718 (https://www.ncbi.nlm.nih.gov/sra/?term=SRR37198718), Biosample SAMN55199529 (https://www.ncbi.nlm.nih.gov/biosample/55199529) and
Bioproject PRJNA1419865 (https://www.ncbi.nlm.nih.gov/bioproject/1419865). The reference datasets used in the present
study are available in the IMG/M system with accessions 3300002231 (https://img.jgi.doe.gov/cgi-bin/m/main.cgi?section=TaxonDetail&page=taxonDetail&taxon_oid=3300002231),
3300009702 (https://img.jgi.doe.gov/cgi-bin/m/main.cgi?section=TaxonDetail&page=taxonDetail&taxon_oid=3300009702), 3300024344 (https://img.jgi.doe.gov/cgi-bin/m/main.cgi?section=TaxonDetail&page=taxonDetail&taxon_oid=3300024344), 3300009703 (https://img.jgi.doe.gov/cgi-bin/m/main.cgi?section=TaxonDetail&page=taxonDetail&taxon_oid=3300009703) for samples RED, V59, VENT and W25,
respectively.

Research involving human participants, their data, or biological material

Policy information about studies with human participants or human data. See also policy information about sex, gender (identity/presentation), and sexual orientation and race, ethnicity and racism.

Reporting on sex and gender	*Use the terms sex (biological attribute) and gender (shaped by social and cultural circumstances) carefully in order to avoid confusing both terms. Indicate if findings apply to only one sex or gender; describe whether sex and gender were considered in study design; whether sex and/or gender was determined based on self-reporting or assigned and methods used. Provide in the source data disaggregated sex and gender data, where this information has been collected, and if consent has been obtained for sharing of individual-level data; provide overall numbers in this Reporting Summary. Please state if this information has not been collected. Report sex- and gender-based analyses where performed, justify reasons for lack of sex- and gender-based analysis.*
Reporting on race, ethnicity, or other socially relevant groupings	*Please specify the socially constructed or socially relevant categorization variable(s) used in your manuscript and explain why they were used. Please note that such variables should not be used as proxies for other socially constructed/relevant variables (for example, race or ethnicity should not be used as a proxy for socioeconomic status). Provide clear definitions of the relevant terms used, how they were provided (by the participants/respondents, the researchers, or third parties), and the method(s) used to classify people into the different categories (e.g. self-report, census or administrative data, social media data, etc.) Please provide details about how you controlled for confounding variables in your analyses.*
Population characteristics	*Describe the covariate-relevant population characteristics of the human research participants (e.g. age, genotypic information, past and current diagnosis and treatment categories). If you filled out the behavioural & social sciences study design questions and have nothing to add here, write "See above."*
Recruitment	*Describe how participants were recruited. Outline any potential self-selection bias or other biases that may be present and how these are likely to impact results.*
Ethics oversight	*Identify the organization(s) that approved the study protocol.*

Note that full information on the approval of the study protocol must also be provided in the manuscript.

Field-specific reporting

Please select the one below that is the best fit for your research. If you are not sure, read the appropriate sections before making your selection.

☐ Life sciences ☐ Behavioural & social sciences ☒ Ecological, evolutionary & environmental sciences

For a reference copy of the document with all sections, see nature.com/documents/nr-reporting-summary-flat.pdf

Ecological, evolutionary & environmental sciences study design

All studies must disclose on these points even when the disclosure is negative.

Study description	The study used marine cores recovered from Santorini, Greece during International Discovery Program Expedition 398. (IODP Exp 398). The study focuses on geochemical and microbiological analysis to reconstruct a hydrothermal past at Santorini caldera. Trace metal and metagenomic analysis were the primary methods used. Two rounds of trace metal analysis was carried out.
Research sample	The research samples were sediment marine cores. The cores, which are collectively called a Hole, were split up into 5 lithological units (Units L1-L5). The samples focused on Site U1595 from IODP Exp 398, which consisted of three holes (A-C). For the metagenomic analysis, DNA was extracted from one sediment sample of the marine core. We choose these cores as they were taken from within the Santorini caldera and exhibit the first recovery of any deep material from the Santorini volcanic system which allows us to better understand the evolution of this complex volcano's hydrothermal system which has currently been under-explored. The samples themselves consisted of unlithified and unmineralized marine sediments.
Sampling strategy	Samples were taken at 20cm intervals throughout the core for consistency, if there was a dramatic change in lithology or strange features within the sediments (colour banding, coarsening etc), extra samples were taken (this only occurred within Unit L3 at Site U1595). Site U1595 reach roughly 130 meters below the seafloor (mbsf). There were some sections, primarily in the lower parts (90-110 mbsf) that had worse recovery than shallow parts and thus fewer samples were obtained. In total 298 samples were analysed for mercury and provided a large and robust dataset. Of those samples, 40 were analysed for trace metals. These 40 were representative of the whole 130 mbsf at Site U1595A. A focus on Unit L3 (55-60 mbsf) was taken as it represented the longest period

	of time and contained the fewest volcanic products compared to other lithological units. Sample sizes for analysis were determined to get a reliable and even spread of samples throughout the core, with emphasis on Unit L3, core recovery permitting. For the metagenomic analysis, only one sample from Unit L3 was analysed as it was the only sample that had sufficient DNA recovery.
Data collection	The lithology, depths and locations of the samples were collected by the shipboard scientists on IODP Exp 398. The geochemistry, timescales, and metal flux data was collected by Sofia Della Sala using a PerkinElmer NexION 350D Inductively Coupled Plasma Mass Spectrometer (ICP-MS) and a LUMEX RA-915M with PYRO-915+ attachment Hg analyser. The metagenomic analysis was collected by Vasiliki Papadimitriou and Paraskevi Polymenakou using an Illumina NovaSeq 6000 S4 PE150 XP.
Timing and spatial scale	The cores were recovered on the expedition in January 2023 and subsampled for analysis in July 2023. Further subsampling was carried out in May 2024. Hg analysis was carried out periodically between October 2023 and November 2025. ICP-Ms analysis was carried out in June and November 2024. Metagenomic analysis was carried out in November 2024. Spatial scale n.a.
Data exclusions	No data were excluded from the analyses.
Reproducibility	For every ICP-MS run and Hg analysis, certified reference materials were used, both as a sample and directly into the machine for quality testing. Multiple standards were used to ensure robustness. In particular for ICP-MS, SLRS-6 was used a a standard to test machine capabilities and BCR-2 was used as a methodology standard to ensure results were reliable. For the Hg analysis NIST 2587 was used. Samples were also analysed on different days to ensure reliable results. Samples were duplicated in both the preparation and on the instrument as well as analysing the same sample months apart to make sure there was no ambiguity in the data. The metagenomic analysis was only able to be carried out on one sample due to low DNA recovery.
Randomization	This is not super relevant to the study as no experiments were conducted to test variables and outcomes, instead analytical chemistry and genomic sequencing was carried out on environmental samples.
Blinding	Blinding is not relevant to the study as no experiments were carried out where the outcome could be prone to bias or misinterpretation due to blinding or randomization. This study focuses on the chemical and metagenomic analysis of environmental sediments from marine cores and no experimental work was done.

Did the study involve field work? ☒ Yes ☐ No

Field work, collection and transport

Field conditions	Marine expedition in January 2023. Full report in IODP proceedings. https://publications.iodp.org/proceedings/398/398title.html
Location	Santorini caldera at Site U1595: 36°22.8955′N, 25°24.3630′E. The three holes at Site U1595, were drilled 50m apart. Seafloor was calculated at 291.6 meters below sea level.
Access & import/export	The project was in collaboration with Greek authorities, with Greek scientists on board the vessel (Paraskevi Nomikou) and with the Greek government's approval by the Hellenic Republic Ministry of Environment. Full permissions were obtained for sample collection in 2022-2023.
Disturbance	Minimal (sea floor coring; see https://publications.iodp.org/proceedings/398/398title.html)

Reporting for specific materials, systems and methods

We require information from authors about some types of materials, experimental systems and methods used in many studies. Here, indicate whether each material, system or method listed is relevant to your study. If you are not sure if a list item applies to your research, read the appropriate section before selecting a response.

Materials & experimental systems

n/a	Involved in the study	
☒	☐	Antibodies
☒	☐	Eukaryotic cell lines
☒	☐	Palaeontology and archaeology
☒	☐	Animals and other organisms
☒	☐	Clinical data
☒	☐	Dual use research of concern
☒	☐	Plants

Methods

n/a	Involved in the study	
☒	☐	ChIP-seq
☒	☐	Flow cytometry
☒	☐	MRI-based neuroimaging

Plants

Seed stocks	*Report on the source of all seed stocks or other plant material used. If applicable, state the seed stock centre and catalogue number. If plant specimens were collected from the field, describe the collection location, date and sampling procedures.*
Novel plant genotypes	*Describe the methods by which all novel plant genotypes were produced. This includes those generated by transgenic approaches, gene editing, chemical/radiation-based mutagenesis and hybridization. For transgenic lines, describe the transformation method, the number of independent lines analyzed and the generation upon which experiments were performed. For gene-edited lines, describe the editor used, the endogenous sequence targeted for editing, the targeting guide RNA sequence (if applicable) and how the editor was applied.*
Authentication	*Describe any authentication procedures for each seed stock used or novel genotype generated. Describe any experiments used to assess the effect of a mutation and, where applicable, how potential secondary effects (e.g. second site T-DNA insertions, mosiacism, off-target gene editing) were examined.*
