# Supplementary material for: A prolonged hydrothermal past at Santorini Caldera revealed by sedimentary trace metal and microbial signatures
Source: Nat Commun. 2026 Aug 4;17:9398. doi: 10.1038/s41467-026-75931-8 (PMC13534618; doi:10.1038/s41467-026-75931-8)
Supplement: Supplementary file 4 [file 41467_2026_75931_MOESM6_ESM.pdf]

nature portfolio

Peer Review File

A prolonged hydrothermal past at Santorini Caldera revealed by sedimentary trace metal and microbial signatures

Corresponding Author: Ms Sofia Della Sala

This file contains all reviewer reports in order by version, followed by all author rebuttals in order by version.

Attachments originally included by the reviewers as part of their assessment can be found at the end of this file.

Version 0:

Reviewer comments:

Reviewer #1

(Remarks to the Author)
I am delighted with this largely successful attempt to resolve the time scale of hydrothermal activity and heavy metal emissions in the Santorini caldera based on deep-sea drilling and coring, and to look for microbial correlates of heavy metal presence, specifically mercury reductase genes. By and large, this manuscript shows that mineralogy and microbiology can be coupled to mutual advantage, and I think the payoff here is much stronger than in a previous effort by other authors (Mara et al. 2024. Communications Earth & Environment 5:505. Doi: 10.1038/s43247-024-01662-7). However, as a microbiologist I am not quite happy with the skimpy documentation of the microbial sequence data (see point 2 below), and with the limited scientific mileage that has been extracted from them (see point 1 below).
1) Are the mercury reductases listed in the sequence table (678130_0_source_data_457588_t5vfgv) actually identical to the mercury reductases of bacterial and archaeal species listed in column E of this table (“Lineage”), or are they only the best available matches in NCBI, and therefore not identical at the nucleic acid or protein sequence level? The microbiology note text does not clarify this problem. Please note that it is exceedingly rare to identify genes obtained in metagenomic sequencing surveys down to the species level. In most cases, genes from a particular bacterial or archaeal species in pure culture have some degree of similarity to the environmental versions of a gene (pulled from diverse natural sample materials), but perfect 1:1 matches with 100% sequence identify are very unusual. To allow readers to check the degree of homology between environmental genes and their NCBI homologues, the sequences in question should be provided in a supplemental file, together with a phylogeny (see for example Ramirez et al. 2021 PLoSOne 16(9): e0256321. https://doi.org/10.1371/journal.pone.0256321). With 39 sequences tabulated, this is perfectly doable. Adding this documentation is a good way to augment and amplify the microbial portion of this study, which is otherwise somewhat slim (too close to the absolute minimum required, and likely to exasperate microbiology readers; I would have liked to use the German word “dürftig” that carries connotations of threadbare and miserly economy. I assume that the bulk of the sequence data will be reported later in a separate publication, but this is no reason for skimping, especially not in a submission to Nature Communications.) While you are at it, do you find other interesting heavy metal reductases? Pinpointing their existence and providing an initial outlook would be helpful, even if a detailed analysis needs to be postponed for another paper.
2) Database submission requirement. Sequence data have to be submitted to, and need to be available from a public database, commonly NCBI (https://www.ncbi.nlm.nih.gov). It appears that sequence data were submitted to the integrated microbial genomes and metagenomes (IMG/M) system at the Joint Genome Institute, but currently no data link to NCBI is provided. Please submit your genomic data to NCBI, and provide a database statement with links to NCBI (and make sure that the links are fully activated and actually work, which takes a little time). This is standard practice for journals and articles that are reporting sequence data.

Further details:
Abstract, lines 52 to 54: ”Geochemical and metagenomic data of sediments record vigorous and pulsatory hydrothermal activity and metal fluxes for ~1100 years, between the caldera-forming Minoan eruption (17th-century BCE), and the major submarine eruption of 726 CE.” This time frame, from approx. 1700 BCE to 726CE, greatly exceeds 1100 years. Please resolve this contradiction.

Supplementary data / Microbiology note: Correct the grammar: “The most dominant species mediating mercury reduction

was …

Reviewer #2

(Remarks to the Author)
The authors give an account of (largely geochemical) results stemming from IODP Expedition 398 to the Santorini caldera, Greece. They have analysed the trace metal content of several cores with an emphasis on Hg and associated microbes, but also mention other metals such as Cu, Mo, Ag, etc.

It is hard to know what the real focus of this contribution is? Seemingly, it is Hg. Its content, flux through time and effect on microbes. But the microbial work seems to be parachuted into the paper and does not connect very well with the trace element data, save that the latter are considered hydrothermally derived and the microbes are associated with that activity. In general, the whole paper does not hang together very well and appeals as more of a report than a scientific contribution. There are several wide sweeping statements made that don't add to the paper and are often inaccurate. The contribution is woefully inadequate with respect to references cited, seemingly not knowing about a myriad of other publications that are germane to this contributon?

There is also inadequate reference to mineralogy, save some SEM microphotographs of barite, etc. What of all the alteration mineral phases? Mineralogy is key to understanding hydrothermal conditions, past and present, and can provide some of the missing information the authors need to support their contention, for example, of previously higher temperature vent fluids (see de Ronde et al., 2019—Geology). Alteration mineral assemblages are the key to unlocking the fluid evolution in these systems.

The authors, in this reviewer's opinion, miss a golden opportunity to add to the current debate on the source of metals such as Cu and Mo to mineralised systems sitting atop submarine caldera volcanoes. The authors calculate some very interesting fluxes for these metals, and others, for the time interval covered by their drill cores. Could this system be a shallow level representation of some sort of hybrid porphyry Cu-Mo system?

In summary, I think the contribution as presented is not focused sufficiently and does not have a logical science thread running through it. It quite possibly is not suited for this journal?

Cornel E.J. de Ronde
ESNZ

Reviewer #3

(Remarks to the Author)
I have attached a separate file with my revisions as well as added comments to the manuscript file itself (word doc).

Version 1:

Reviewer comments:

Reviewer #1

(Remarks to the Author)
Thank you for responding to my criticism by doing additional analyses on heavy metal-processing genes; the microbiology content of the manuscript is significantly improved and I have no further comments.

Reviewer #2

(Remarks to the Author)
I have reviewed, for the 2nd time, the ms "A prolonged hydrothermal past at Santorini Caldera revealed by sedimentary trace metal and microbial signatures" by Della Sala et al. The ms is much improved and indeed the text on both the metal fluxes and the microbes is much tighter. However, my overriding comment is similar to what I said in my previous review. That is, the connection between the hydrothermal results and metal fluxes, and the microbial work, appears disjointed and not that strong? The work on the stratigraphy of the upper parts of the caldera floor, the time represented by the sediments, the trace element geochemistry and fluxes is good. So too the description of the microbes present and what they might mean. But the connection between the two different pieces of work is not compelling. I feel both (and especially the flux work) could stand alone whereas together there is not a seamless connection? It still very much feels like two separate pieces of work slammed together? What is the main relevance of the microbial population described? How is this important?

Some minor observations after reading the revised ms again:

1. Henley et al. (2024—JVGR) is a good reference with regards discolouration of seawater surrounding a submarine volcanic edifice as a precursor to eruption.

2. Explain the dashed lines in Fig 2a in the figure caption (presumably no data?).

3. Lines 201-203. You can get laterally 'uniform' enrichments in the metals in the caldera floor due to metal deposition via plumes in the water column. Given a stated heterogeneous distribution of metals over the caldera floor, perhaps that reflects seperate subseafloor discharge areas?

4. Line 256. "polymetallic hydrothermal activity" is an awkward phrase. You mean 'hydrothermal activity associated with polymetailic mineralization'.

5. Are the authors aware of the microbial work done by Reysenbach et al. (2020—PNAS) associated with seafloor hydrothermal vents at Brothers volcano? Might provide further insight and/or comparison.

6. Line 280. "...pulsing of hydrothermal activity..." is described in some detail by de Ronde et al. (2005; 2011) for Brothers volcano of the Kermadec arc over a range of time scales from data related to short term vent fluid chemistry, sulphide isotopes, etc. Similalry, Stucker et al. (2023—Econ. Geol.) for vent fluids over a greater time interval, and Walker & de Ronde (2023—Econ. Geol.) for hydrothermal plumes over the volcano. All point to 'pulsed' contributions to the hydrothermal system, or at least episodes of more (or less) vigouous activity, especially w.r.t. magmatic contributions. Is this the link between the work done by the authors of metal fluxes and the microbes??

7. Line 325. I would agree with this statement only it appears to contradict a previous statement in line 202?

8. Line 335. This sentences corroborates the work done by de Ronde et al. (2005; 2011) and others have said same to have occurred at Brothers volcano.

9. Line 343. You have a 'z' in the formula but refer to 'h' in the text for depth down the core. Be consistent.

In summary, the ms is much tighter and better overall, but I struggle with the point of the microbial work seemingly juxtaposed against the metal flux work. There are no statements of how the former must be due to the other, or is reliant on the other, or indeed, what relevance are the microbes overall in this study on metal flux? What difference does it make to our world to have these types of microbes on the seafloor?

Reviewer #3

(Remarks to the Author)
The authors have addressed my concerns comprehensively and the manuscript has been substantially improved. The revised version is now more cohesive and better aligned with the main ideas of the manuscript. The different sections are integrated much more effectively throughout the paper, including the microbiology, overall improving the flow and readability. The data was further integrated into a broader context.
Importantly, the questions regarding the methods and sampling strategy have been addressed and the methodological transparency has improved.

As a minor remaining comment, I still think that the discussion of the two possible hydrothermal models (more widespread low-temperature diffuse venting vs. higher-temperature focused venting) remains somewhat vague. At the moment, the concept is being introduced and discussed very briefly.
The authors state that this cannot currently be resolved without additional data, which is reasonable.
However it would help the reader if the authors could elaborate slightly further on what processes or controls may favors these two styles of venting and whether the current observations lean more towards one interpretation (for example based on implications from other hydrothermal systems). In this context, the conceptual model shown in Fig. 5 could also be integrated a bit more strongly into the main text. That said, I understand that this is not the primary focus of the manuscript.

Please note that there are still a few small cosmetic issues throughout the manuscript (minor typos, missing bracket, etc.) that should be corrected before publication.

Open Access This Peer Review File is licensed under a Creative Commons Attribution 4.0 International License, which permits use, sharing, adaptation, distribution and reproduction in any medium or format, as long as you give appropriate credit to the original author(s) and the source, provide a link to the Creative Commons license, and indicate if changes were made.
In cases where reviewers are anonymous, credit should be given to 'Anonymous Referee' and the source.
The images or other third party material in this Peer Review File are included in the article's Creative Commons license, unless indicated otherwise in a credit line to the material. If material is not included in the article's Creative Commons license and your intended use is not permitted by statutory regulation or exceeds the permitted use, you will need to obtain permission directly from the copyright holder.
To view a copy of this license, visit https://creativecommons.org/licenses/by/4.0/

We thank the editor and reviewers for their comments, which have really helped us to produce a better manuscript.

We appreciate Reviewer 1's enthusiasm for the integration and connection between microbiology and geology, which we have further improved with these revisions. Reviewer 2's interest in the metals specifically has enabled us to expand the scope of the manuscript, and we were grateful for Reviewer 3's confidence in the conclusions and implications.

Our revisions have expanded and reworked the microbiology section, considered several other metal systems, and incorporated new information on metal fluxes and budgets. We have expanded the scope of the paper, integrated the microbiology better within the manuscript and made comparisons with other systems around the world.

Please see below a point-by-point reply of the suggested revisions (in numerical order R1-R3):

Reviewer 1

Reviewer 1 comment 1: *However, as a microbiologist I am not quite happy with the skimpy documentation of the microbial sequence data (see point 2 below), and with the limited scientific mileage that has been extracted from them (see point 1 below).*

1) *Are the mercury reductases listed in the sequence table (678130_0_source_data_457588_t5vfgv) actually identical to the mercury reductases of bacterial and archaeal species listed in column E of this table ("Lineage"), or are they only the best available matches in NCBI, and therefore not identical at the nucleic acid or protein sequence level? The microbiology note text does not clarify this problem…To allow readers to check the degree of homology between environmental genes and their NCBI homologues, the sequences in question should be provided in a supplemental file, together with a phylogeny (see for example Ramirez et al. 2021 PLoSOne 16(9): e0256321. https://doi.org/10.1371/journal.pone.0256321). With 39 sequences tabulated, this is perfectly doable. Adding this documentation is a good way to augment and amplify the microbial portion of this study…While you are at it, do you find other interesting heavy metal reductases? Pinpointing their existence and providing an initial outlook would be helpful, even if a detailed analysis needs to be postponed for another paper.*

Author reply: We appreciate these suggestions, which help strengthen the microbiological component of the paper.

Regarding the taxonomic affiliation of the mercury reductase genes, we need to clarify that the genes listed in the sequence table are not claimed to be identical (100% nucleotide or amino acid identity) to cultured representatives of the bacterial or archaeal species shown in the "Lineage" column. Instead, the lineage assignment corresponds to the best available matches based on the IMG/M annotation pipeline.

The metagenome U1595C was annotated within the IMG/M system (Integrated Microbial Genomes & Microbiomes, DOE-JGI) using its standard pipeline. Mercury reductase genes were retrieved using the KEGG Orthology identifier K00520 (mercuric reductase), yielding 39 gene hits as you can see in the screenshot below from IMG/M system. For each of the genes the following information is provided in the IMG/M platform including the IMG NR Genome, evidence for Function Prediction together with External Sequence search in NCBI (see screenshot below).

KEGG Orthology (KO) (assembled)

Genome: [Deep subsurface marine sediment microbial communities from Santorini caldera, Hellenic Volcanic Arc, Aegean Sea, Greece - 1595C (*) (MER-FS) (assembled)]

List Genes | Add Selected to Gene Cart | Add Selected to Function Cart | Select All | Clear All

Filter column: All Columns Filter regex : K00520 Apply

Export Page 1 of 1 << first < prev 1 next > last >> All

Column Selector Select Page Deselect Page

Select	KO ID	Name	Definition	Gene Count
☐	KO:K00520	merA	mercuric reductase [EC:1.16.1.1]	39

Export Page 1 of 1 << first < prev 1 next > last >> All

List Genes | Add Selected to Gene Cart | Add Selected to Function Cart | Select All | Clear All

Gene Information

Gene Information	
Gene ID	Ga0618213_000024_22893_24587
Gene Symbol	
Locus Tag	null
Product Name	mercuric reductase
IMG Product Source	KO:K00520
Assembled/Unassembled	assembled
Genome	Deep subsurface marine sediment microbial communities from Santorini caldera, Hellenic Volcanic Arc, Aegean Sea, Greece - 1595C
DNA Coordinates	22893..24587 (+)(1695bp)
Scaffold Source	Ga0618213_000024 (356047bp)
GC Content	0.66
Estimated Copy	204
**IMG NR Best Hit**	
IMG NR Gene	2624906278 mercuric reductase
IMG NR Genome	2623620556 Dyella jiangningensis SBZ 3-12<br>(Lineage: Bacteria;Pseudomonadota;Gammaproteobacteria;Lysobacterales;Rhodanobacteraceae;Dyella;Dyella jiangningensis)<br>(GTDB Lineage: d__Bacteria;p__Pseudomonadota;c__Gammaproteobacteria;o__Xanthomonadales;f__Rhodanobacteraceae;g__Dyella;s__Dyella jiangningensis_B)
Percent Identity	100 %
**Protein Information**	
Amino Acid Sequence Length	564aa
UniProt Search	The gene search against UniProt (https://www.uniprot.org/)<br>feature is a collaboration work with KBase (https://www.kbase.us/). Database version: UniRef90 Release 2020_02<br>Search (Search will open in a new window.)
COG	COG1249 - Pyruvate/2-oxoglutarate dehydrogenase complex, dihydrolipoamide dehydrogenase (E3) component or related enzyme
Families	- TIGR02053 mercury(II) reductase (bit_score=616.3)
Pfam	pfam00403 - HMA<br>pfam02852 - Pyr_redox_dim<br>pfam07992 - Pyr_redox_2
SUPERFam	55424 - FAD/NAD-linked reductases, dimerisation (C-terminal) domain<br>51905 - FAD/NAD(P)-binding domain
CATH FunFam	3.30.390.30 - unclassified<br>3.30.70.100 - unclassified<br>3.50.50.60 - FAD/NAD(P)-binding domain

Evidence For Function Prediction

Neighborhood

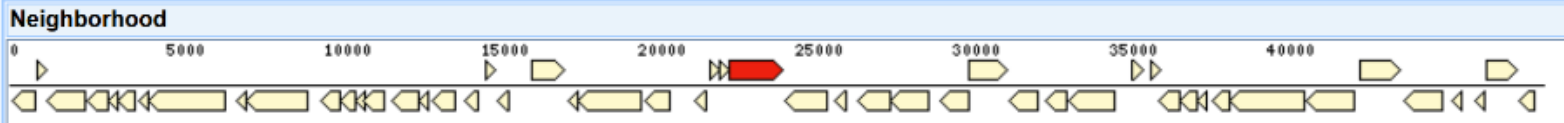

red = Current Gene
CRISPR array
Sequence Viewer For Alternate ORF Search

Conserved Neighborhood

Show neighborhood regions with the same top isolate hit

COG

COG ID	Consensus Sequence Length	Description	Percent Identity	Alignment On Query Gene	E-value	Bit Score
COG1249	454	[C] Energy production and conversion<br>Pyruvate/2-oxoglutarate dehydrogenase complex, dihydrolipoamide dehydrogenase (E3) component or related enzyme	98.9		4.0e-128	432
COG2608	71	[P] Inorganic ion transport and metabolism<br>Copper chaperone CopZ	90.41		7.6e-10	41

Pfam

Pfam Domain	Pfam Hit	Description	Percent Alignment On Query Gene	Alignment On Query Gene	E-value	Bit Score
HMA	pfam00403	Heavy-metal-associated domain	11.17		1.2e-11	49
Pyr_redox_dim	pfam02852	Pyridine nucleotide-disulphide oxidoreductase, dimerisation domain	19.50		3.5e-27	99
Pyr_redox_2	pfam07992	Pyridine nucleotide-disulphide oxidoreductase	56.74		4.3e-62	214

External Sequence Search

NCBI BLAST
EBI InterPro Scan
Protein Data Bank BLAST

In the original submission, our intention was to demonstrate the taxonomic diversity of microorganisms potentially involved in mercury reduction, rather than to present species-level identity. We agree with the Reviewer that species-level resolution in metagenomic datasets is rarely achievable. In the revised manuscript, we performed additional analysis for key metal resistance and metal transport genes (i.e. for Mn, Fe, Mo, Cu, Co-Zn-Cd, As) and an updated Supplementary Figure 2 and Supplementary Figure 3 are now presented. We also appreciate the reviewer's suggestion to perform phylogenetic reconstruction similar to Ramirez et al. (2021). However, although we acknowledge the value of such analysis, the study by Ramirez et al. was based on targeted amplicon sequencing, while our study is based on shotgun metagenomic sequencing which consists of fragmented genomic sequences derived from multiple taxa and not near full length marker gene sequences as the amplicons.

In addition, we need to emphasize that metagenomic analysis in highly oligotrophic and diverse environments such as those in the Aegean Sea is a challenging task. In our case, we used a specialized lab protocol and the sample was processed multiple times in order to ensure that high quality and high molecular weight DNA was extracted for metagenomic analysis. These datasets are limited, unique and valuable allowing us to understand microbial functional potential in oligotrophic and volcanic Mediterranean environments. These points are highlighted in the revised version of the manuscript. For example, in line 259, we have added: "Recovering high molecular weight DNA from deep subsurface sediments is a challenging task due to low biomass and DNA degradation with depth and time."

Regarding the additional heavy metal reductases: While our initial focus was on mercury-related genes due to the elevated Hg concentrations and their relevance to past hydrothermal activity, we further screened the metagenome for additional metal-related genes. Beyond mercury reductases, we identified genes associated with iron reduction and arsenate reductase. In addition, we discuss their potential geochemical relevance. We have rewritten the microbiology section, beginning with: "The results reveal a diverse repertoire of genes encoding proteins responsible for resistance to toxic metals. Most metals are essential to the function of organisms but in elevated concentrations they can disrupt the homeostasis and become toxic for marine microorganisms. We identified systems offering resistance or adaptation to elevated levels of Hg, As, Cu, Mn, Fe, Mo, Co, Zn and Cd. These mechanisms are key for metal cycling in metal-enriched environments."

We then go further to discuss the metal-adapted systems and their significance in contributing to the evidence of a hydrothermal past at Santorini. For example:

"These data support the interpretation that Unit L3 formed during metal-enriched hydrothermal conditions. In particular, high abundances of *czc*A (3.44), *cop*A (3.75), *mnt*H (0.737) and *feo*B (2.36) along with elevated levels of *ars*C (0.98) and *mer*A (0.41) in site U1595 compared to the W25 background (0.14, 0.58, 0, 0.21, 0.38, 0.07 respectively) (Fig.3a) are consistent with a prolonged exposure to polymetallic hydrothermal activity. The prevalence of efflux systems (*czc* and *cop*) implies a strong selective pressure for detoxification and intracellular metal homeostasis. The low levels of *mtr*A genes (0.67 and 0.49 in site U1595 and W25, respectively indicate that Fe^{3+} reduction was not a dominant pathway, potentially due to the fact Fe was mostly present as dissolved Fe^{2+} in hydrothermal fluids rather than particulate Fe^{3+} oxides. This is supported by the increased *feo* levels which suggests bioavailable Fe^{2+} under reducing conditions—typical of hydrothermally influenced sediments."

<u>Reviewer 1 comment 2</u>: *2) Please submit your genomic data to NCBI, and provide a database statement with links to NCBI (and make sure that the links are fully activated and actually work, which takes a little time). This is standard practice for journals and articles that are reporting sequence data.*

Author reply: In addition to IMG/M Data Management System which we routinely use for our data, and will be open to the public upon publication, we followed the recommendation by Reviewer 1 and submitted the raw data to the NCBI. A related statement has been also added to the revised version of the manuscript. Specifically, the following text was added into the Data Availability section of the revised version of the manuscript:

"The deep subsurface sequence reads of the present study (U1595C Unit L3) have been deposited to the IMG/M system with accession 3300066567 and to the National Centre for Biotechnology Information Sequence Read Archive under access numbers SRR37198718, Biosample SAMN55199520 and Bioproject PRJNA1419865.

Reviewer 2

Reviewer 2 comment 1: *The microbial work seems to be parachuted into the paper and does not connect very well with the trace element data, save that the latter are considered hydrothermally derived and the microbes are associated with that activity.*

Author reply: We appreciate that there seemed to be a disconnect between the microbiology and the geology. We have further integrated the microbiology portion, particularly the evidence for genomic adaptations to potentially toxic metals, with the trace metal enrichment profiles. By analysing for genomic adaptations to the same metals as in the geochemistry section, these two sections are now much more closely linked. We have also made an effort to refer more directly to the microbiology aspect in the introduction as well as refer back to the geochemical data within the microbiology section itself to better integrate these lines of evidence. In the Introduction we added:

"Submarine hydrothermal systems provide a distinctive environment for microbial communities and microbial activity is known to play an essential role in the cycling of iron in the shallow hydrothermal embayments[44,45] around the Kameni Islands[30]."

In addition, within the microbiology section, we have added this that refers back to the geochemical section:

"Sediment concentrations of Mn (mobile under reducing conditions) and Mo (immobile under sulfidic conditions) peak at different depths in the L3 sediments (Fig. 2b), providing further evidence for reducing conditions and redox fluctuations. Collectively, this gene distribution reflects the nature of the fluids and sediments at the time of deposition, and supports the interpretation of the hydrothermal origin of the trace metals in Unit L3."

Reviewer 2, comment 2: *There is also inadequate reference to mineralogy, save some SEM microphotographs of barite, etc. What of all the alteration mineral phases? Mineralogy is key to understanding hydrothermal conditions, past and present, and can provide some of the missing information the authors need to support their contention, for example, of previously higher temperature vent fluids (see de Ronde et al., 2019—Geology). Alteration mineral assemblages are the key to unlocking the fluid evolution in these systems.*

Author reply: The sediments we have studied are unconsolidated and unmineralized, and thus the detailed mineralogical work requested (e.g. the alteration mineralogy, or studies of mineralised rocks) is outside the scope of this work – we don't have any such samples from IODP 398. We have now clarified this in the paper at various points:

"Five units (L1–L5) were described shipboard[47,48], all of which were unlithified."

“Concentrations of selected trace elements, considered likely to be associated with hydrothermal activity[50] (i.e. Hg, Sb, As, Mn, Mo, V, Fe, Co, Cu, Pb, Ag), were analysed in bulk samples of the unconsolidated and unmineralized sediments from Holes U1595A (L1–L4) and U1595B (L5).”

Site U1595 (which is the focus of the paper), is considered a distal site within the flooded caldera, as there are no known vent fields nearby. These distal sediments record the trace-metal signatures from past hydrothermal plumes which have influenced the sea-water chemistry of the caldera. We have clarified this point in the manuscript and methods. In the Evolution of the pre-726 CE hydrothermal system section (currently just under Fig. 4) we added the following:

“Distal basin-floor sites can give basin-scale information about the influence of hydrothermal inputs into the flooded caldera, as the metal-rich hydrothermal plumes rise from the submarine vents, entrain and mix with ambient seawater, and their metals are scavenged by Fe and Mn oxides[36]. Co-enrichment of Hg with Fe and Mn is indicative of a hydrothermal plume settling out into the sediments, which is consistent with our data (Fig. 2 and Fig. 4). In both the Central Indian Ridge and Kagoshima Bay in Japan, hydrothermal Hg was detected 1-10 km away from active vent sites[35,36]. ”

<u>Reviewer 2, comment 3:</u> *The authors, in this reviewer's opinion, miss a golden opportunity to add to the current debate on the source of metals such as Cu and Mo to mineralised systems sitting atop submarine caldera volcanoes. The authors calculate some very interesting fluxes for these metals, and others, for the time interval covered by their drill cores. Could this system be a shallow level representation of some sort of hybrid porphyry Cu-Mo system?*

<u>Author reply:</u> We appreciate the Reviewer’s interest in the source of metals, in particular Cu and Mo, in ‘mineralised systems sitting atop submarine caldera volcanoes’ but we emphasise that this is not the geological context of this paper. Site U1595 is unmineralized and distal, and, due to its relatively shallow recovery, the drilling did not reach any epithermal deposits. We agree that metal deposition fluxes are of relevance, and we have moved some information which was previously in the supplementary to the main text (now Table 3 in the manuscript), whereby we detail the fluxes and budget for a suite of metals of interest. We have included and expanded the discussion around this section to broaden the scope of the paper and highlight the importance of metals such as Cu and Mo in the system. We write:

“We see similar trends for the other hydrothermally derived trace metals (Table 3) with Mn, V, Co, Cu, As, Mo, Sb, Pb and Ag all exhibiting a flux in Unit L3 an order of magnitude larger than in L1. The quantities of metals deposited within the caldera-floor sediments during the L3 hydrothermal event included 5900 tonnes of Cu, 1620 tonnes of Mo and 16 tonnes of Ag.”

We also compare these results to other systems, in particular New Zealand, which Reviewer 2 referred to in their in-text comments:

“The deposition fluxes of Ag, Hg, Sb and As that we estimate for Unit L3 are similar to the hydrothermal fluid fluxes estimated for the present-day Broadlands-Ohaaki and Rotokawa geothermal fields in Taupo, New Zealand[10]. This comparison confirms that submarine arc volcanoes can also be significant sources of hydrothermal metal enrichment, and of metal emission to the hydrosphere and atmosphere. By analogy with the Taupo volcanic system, we would expect that the sustained release of metals from Santorini hydrothermal vents since the caldera-forming eruption 3500 years ago is the sea-floor expression of a shallow crustal epithermal system, which has the potential to concentrate and deposit metals.”

Reviewer 3

Reviewer 3 comment 1: Generally, I think the different sections of the manuscript could be better related and aligned, for example the abstract, introduction and conclusion. From the abstract, I expected a bigger focus on the dynamics of the paleo-hydrothermal system and how it relates to metal enrichments. The introduction then brings up different tectonic settings where hydrothermal systems occurs, however they are not further discussed in regard to the results of the manuscript. The metal resources are barely mentioned there. The conclusion and the final model (Fig. 5) then suddenly focuses on different possible conceptual models of the paleo hydrothermal system, which were neither mentioned in the abstract nor in the introduction.

Author reply: We have put more emphasis on the dynamics of the paleo-hydrothermal system in the introduction and added the different conceptual models for the paleo-system in the introduction as well as throughout the results, so that the manuscript is more cohesive and well-aligned. In the introduction, we added:

"Previous work within the Santorini caldera has been restricted to sampling the youngest sea-floor sediments from short cores ranging from <60 to <250 cm[42,18]. These analyses showed that caldera sediments were influenced by hydrothermal activity, since mean concentrations of Mn, Fe, Cu, Zn, Pb and P were higher in intracaldera sediments than outside the caldera. The cores contained distinct Mn and Fe-enriched layers at depth, which were considered to represent a hydrothermal past that was considered 'more active' than the present day, whether from higher-temperature venting, carrying more metals to the seafloor, or an increase in active vents within the caldera."

This text section also sets out the two scenarios which we refer back to with our conceptual model.

We have also expanded the discussion around metal enrichments with further comparisons to the literature to strengthen the conclusions. We have added in Table 1, which compares certain metal concentrations found in our cores to previous values in the literature. For example, we have added:

"This enrichment is considered to be related to hydrothermal activity, as it is not laterally uniform throughout the caldera, which would be expected if anthropogenic effects dominate[18]. As and Sb have been measured in the hydrothermal sediments of hot-spring embayments of the Kameni Islands, with values of 927 ppm As and 3.21 ppm Sb[28]. In Unit L3 at site U1595, we find maximum values of 1030 ppm As and 4.9 ppm Sb, higher than active hydrothermal sediments in the present-day caldera."

Similarly, we have continued the comparison of tectonic settings throughout the discussion to streamline the manuscript which also helps place the results in a wider context as R3 suggests. We have written:

"The only other submarine Hg flux that has been determined for a subduction zone hydrothermal system is in the Tonga Arc[40], which yielded a total hydrothermal Hg emission flux of 35 kg yr^{-1}, about ~3× higher than the average for Santorini's paleo-hydrothermal system. For a mid-ocean ridge setting, the annual Hg flux from TAG in the Mid-Atlantic Ridge is estimated at a maximum of 4.5kg yr^{-1}, which is comparable with our flux ranges at Santorini…Based on our limited data, we suggest that hydrothermal systems in subduction zones are significant point sources of Hg release to the oceans."

Reviewer 3 comment 2: Additionally, the section regarding the microbiology is not very well connected to the geology…I think it should be better integrated and combined with the geology if possible.

Author reply: We have now included more background to the microbial work in the introduction to provide a better link throughout the paper, but also set it up in context and made the reason for its inclusion in this manuscript clear. We have added:

"Shotgun metagenomic sequencing enabled screening for genes involved in metal resistance and redox transformations. This can help to reveal the seafloor conditions at the time of deposition during past hydrothermal events."

We have also expanded the microbial work to include other trace metals which better ties it to the previous section where we discuss metal enrichments in the sediment cores. In the microbiology section, we have added:

"We identified systems offering resistance or adaptation to elevated levels of Hg, As, Cu, Mn, Fe, Mo, Co, Zn and Cd. These mechanisms are key for metal cycling in metal-enriched environments. Notably, the *mer*A gene from the Hg resistance system, coding for the mercury II reductase protein, which converts organic-bound Hg^{2+} to volatile Hg^0, was present [29,53] (Fig.3a and Supplementary Fig. 2). High levels of *mer*A is consistent with a selective pressure from dissolved Hg^{2+} [53]. Similarly, arsenate reductase genes (*ars*C)[54], copper resistance and efflux genes (*cop*A, *cus*A, *cus*B)[55], and the Co/Zn/Cd efflux system (*czc*A-D)[56] all selectively remove or detoxify the metal they are specific for due to increased exposure from those metals." And also: "These data support the interpretation that Unit L3 formed during metal-enriched hydrothermal conditions. In particular, high abundances of *czc*A (3.44), *cop*A (3.75), *mnt*H (0.737) and *feo*B (2.36) along with elevated levels of *ars*C (0.98) and *mer*A (0.41) in site U1595 compared to the W25 background (0.14, 0.58, 0, 0.21, 0.38, 0.07 respectively) (Fig.3a) are consistent with a prolonged exposure to polymetallic hydrothermal activity…The low levels of *mtr*A genes (0.67 and 0.49 in site U1595 and W25, respectively) indicate that Fe^{3+} reduction was not a dominant pathway, potentially due to the fact Fe was mostly present as dissolved Fe^{2+} in hydrothermal fluids rather than particulate Fe^{3+} oxides. This is supported by the increased *feo* levels which suggests bioavailable Fe^{2+} under reducing conditions—typical of hydrothermally influenced sediments[61]. Sediment concentrations of Mn (mobile under reducing conditions) and Mo (immobile under sulfidic conditions) peak at different depths in the L3 sediments (Fig. 2b), providing further evidence for reducing conditions and redox fluctuations."

Reviewer 3 comment 3: The introduction in general lacks some information relevant to the manuscript. For example, the final conceptual model compares the active system with the paleosystem, so the introduction could include the current hydrothermal activity at Santorini, what are the temperature ranges, what are the metal contents and estimations, precipitation conditions, what minerals (ores) occur ect? This is also interesting in respect to the metal budget and modelling of fluxes. One of the final conclusions refers to low- vs high temperature venting and therefore should be explained in the introduction. I have also added some comments to the manuscript.

Author reply: We have expanded the introduction to provide more context on the research done already at Santorini and what is known about the hydrothermal system, both in terms of the fluids and sediment enrichments. In the Introduction, we have added:

"Investigation of the hydrothermal system in Santorini caldera found that fluids sampled between 1982 and 1997 from the Kameni Island embayments exhibited, on average, a stable chemical composition[25]. Compared with other hydrothermally-active centres along the South Aegean Volcanic Arc, these fluids were the most concentrated in Mn and Fe, reaching a thousand-fold enrichment over normal seawater[7,25]. While there is little evidence for variation on decadal time-scales during a period of volcanic quiescence, analysis of records over longer time-scales is essential to understand the

potential links between deeper magmatic supply, volcanic unrest or eruption and changes in hydrothermal activity."

We have also mentioned the conceptual model and tied it in with existing hypotheses (mentioned this also in reply to comment 1 by Reviewer 3), as well as made clear the scientific knowledge gap we are filling with this study, with a few changes to the end of the introduction section:

"The volcano-sedimentary sequence that was recovered provides a continuous geological archive spanning the last ~3500 years, including the deposits of a major submarine eruption (726 CE eruption)[47] and allows us to track the trace metal and microbial signatures of hydrothermal activity within the Santorini caldera over this same time period. Here we show how the combined analysis of both microbial and sedimentary archives provides evidence for past hydrothermal activity at Santorini."

<u>Reviewer 3 comment 4</u>: *Regarding the sampling and methods. It seems to me that the lower parts of the drill cores, so L4 and L5, are underrepresented in the sampling. I understand that the recovery appears to be worse, however based on the recovery map (in black), there are quite some recoveries also in the deeper parts. However for the trace element analysis, not all of them were selected and I wonder why. This results in quite a big sampling gap at depth. This should be explained somewhere. Vice versa, why was the depth of 55-60 mbsf so strongly sampled?*

<u>Author reply:</u> This observation is indeed correct, we should have specified that in Hole U1595A we only have samples reaching a maximum of 90mbsf and we only had samples from Hole U1595B from 108 to the end of the hole so between 90mbsf and 108 there are no samples even if the core log showed some recovery. We have now clarified this in the manuscript and figure legends. The analysis was conducted in a few stages. Preliminary sampling involved a 'representative' down-core Hg analysis, making sure we took a few samples from each lithological unit, in particular if there was a change in lithology (this predominantly happened in Unit L3 hence the seeming over-representation of that unit). We then conducted a first set of ICP-MS analyses, with a slight focus on Unit L3 since that was the least volcaniclastic unit and showed the most mud layers and heterogeneous lithologies. In addition, L3 represents a longer period of deposition compared to the over and underlying units (instantaneous deposition), its chemistry is likely to be more variable and requires a larger sample set to characterise.

Due to its lithology (tuffaceous mud layers), L3 is more suitable for preserving hydrothermal signals compared to volcanic layers, so a larger focus on Unit L3 was established at this point. However, in subsequent ICP-MS and Hg analyses, we also continued to analyse samples outside of Unit L3 for scientific completeness and rigour. For clarity on this point, we have added the following into the methods:

"A slightly larger sampling focus was put onto Unit L3 following the Hg results and, due to lithology, L3 is more suitable for preserving hydrothermal signals compared to volcanic layers."

We ensured that high-resolution sampling and analysis were conducted for Hg (since this is conducted via a different method), therefore preventing any significant sampling gaps, and this corroborates the trace-metal data and allows us to look into detail into the evolution of the system.

<u>Reviewer 3, comment 5</u>: *Also as this is a hydrothermal vent field, the manuscript would benefit from something in the method about which part of the sediments were used for the trace element analysis.*

<u>Author reply:</u> We have now specified in the methods what constitutes a sample. We have added the following into the methods:

"The cores were sampled at specified locations or intervals, keeping as best as possible to a homogenous lithology, with a couple of grams of material taken per sample. Samples were taken with a standard 1cm-wide plastic 5 cm^3 scoop, and all analyses were conducted on bulk sediment."

In other sections of the manuscript, we have mentioned the lithology of the samples and cores and used the lithologies to contextualise the metal enrichments, so how the samples and their metal contents differ throughout the core, should be addressed. Similarly, we had added in some sections on metal deposition specifying distance from vents and how that might affect enrichments. Just after Fig. 4, we have written:

"Distal basin-floor sites can give basin-scale information about the influence of hydrothermal inputs into the flooded caldera, as the metal-rich hydrothermal plumes rise from the submarine vents, entrain and mix with ambient seawater, and their metals are scavenged by Fe and Mn oxides[36]. Co-enrichment of Hg with Fe and Mn is indicative of a hydrothermal plume settling out into the sediments, which is consistent with our data (Fig. 2 and Fig. 4). In both the Central Indian Ridge and Kagoshima Bay in Japan, hydrothermal Hg was detected 1-10 km away from active vent sites[35,36]."

<u>Reviewer 3, comment 6:</u> *Regarding the evolution of the hydrothermal system I have a few remarks and have also included some in the manuscript. I had trouble to understand how the three phases of hydrothermal activity were defined. There are more than the 6 peaks throughout the depth, there are not three known eruptions but two, and they do not correlate with the cold/wet periods. Was Hg used as a threshold? It would really help the reader to better understand how these phases were defined, so I would suggest to include this in the manuscript. I was also missing a discussion on the shallower peaks? I think the MS would additionally benefit from a comparison to some literature data here to understand what the sediment signature is, the ocean floor signature and the hydrothermal enrichment. The introduction mentions some literature values of Hg in other hydrothermal systems and it would be nice to have this comparison here. I have also suggested in the MS to add some of these references to the Fig. 4.*

<u>Author reply:</u> These have now been specified in the manuscript so hopefully should provide a better understanding. Hg was indeed used as a threshold - we wanted to define the phases irrespectively of eruptions and climatic conditions so as not to introduce bias. So, we identified peaks and troughs within the Hg data and grouped them sensibly. Once the phases were defined, we then correlated them with known eruptions and cold/wet periods to see how they interacted. We have specified:

"On this basis, Phase 1 (pre-phase) lasts <50 years and shows a maximum Hg ~60 ppb, Phase 2 (main phase) collectively lasts 500–1000 years, and has Hg >80 ppb; while Phase 3 (post-phase) lasts 50–100 years with Hg between 40 and 60 ppb. Altogether, this implies a maximum of ~1100 years of heightened hydrothermal activity during the 2300 years of Unit L3."

We have now better included discussion on the more speculative enrichments too: "There may be another pulse of hydrothermal activity corresponding with the eruption in 46–47 CE, that also occurs during a cold/wet period, although this inference is only supported by a single-point increase in Hg."

We have also included literature comparisons into the discussion to strengthen the manuscript as R3 suggests. We have added:

"In both the Central Indian Ridge and Kagoshima Bay in Japan, hydrothermal Hg was detected 1-10 km away from active vent sites[35,36]. Surface sediments proximal to the hydrothermal field in Paleochori

Bay, Milos (neighbouring island to Santorini), show Hg concentrations of ~200 ppb, roughly the same values we observe in our distal sediments prior to 726 CE."

And (see also reply to R3 comment 1):

"The only other submarine Hg flux that has been determined for a subduction zone hydrothermal system is in the Tonga Arc[40], which yielded a total hydrothermal Hg emission flux of 35 kg yr^{-1}, about ~3× higher than the average for Santorini's paleo-hydrothermal system. For a mid-ocean ridge setting, the annual Hg flux from TAG in the Mid-Atlantic Ridge is estimated at a maximum of 4.5kg yr^{-1}, which is comparable with our flux ranges at Santorini…Based on our limited data, we suggest that hydrothermal systems in subduction zones are significant point sources of Hg release to the oceans."

<u>Reviewer 3, comment 7:</u> The integration of some literature data would increase the significance of this manuscript by giving the reader some context to other hydrothermal systems. I would suggest to include the tectonic setting into the discussion.

<u>Author reply:</u> We have expanded the discussion and included comparisons with other systems, and have also included some discussion around other metals, namely Ag, As and Sb to also support that data and how it compares globally. We have added:

"The deposition fluxes of Ag, Hg, Sb and As that we estimate for Unit L3 are similar to the hydrothermal fluid fluxes estimated for the present-day Broadlands-Ohaaki and Rotokawa geothermal fields in Taupo, New Zealand[10]. This comparison confirms that submarine arc volcanoes can also be significant sources of hydrothermal metal enrichment, and of metal emission to the hydrosphere and atmosphere. By analogy with the Taupo volcanic system, we would expect that the sustained release of metals from Santorini hydrothermal vents since the caldera-forming eruption 3500 years ago is the sea-floor expression of a shallow crustal epithermal system, which has the potential to concentrate and deposit metals."

And:

"Our deposition flux estimates for Fe and Mn for the past ~400 years within Unit L1 are 111 t yr^{-1} (Fe) and 2.61 t yr^{-1} (Mn); these compare well with earlier estimates[42] (88.9 t yr^{-1} Fe and 3.85 t yr^{-1}, adjusted for measured bulk sediment density."

For the comparison to other hydrothermal systems in Greece and to contextualise our results, we have added:

"Compared with other hydrothermally-active centres along the South Aegean Volcanic Arc, these fluids were the most concentrated in Mn and Fe, reaching a thousand-fold enrichment over normal seawater[7,25]."

And:

"Surface sediments proximal to the hydrothermal field in Paleochori Bay, Milos (neighbouring island to Santorini), found Hg concentrations of 183.9 ppb and 243.3 ppb, roughly the same values we observe in our distal sediments prior to 726 CE."

<u>Reviewer 3, comment 8:</u> Lastly, this manuscript is mainly working with samples from one drill site and I would suggest to add a remark –whether in the manuscript or the supplement- about the variations

across the caldera. To understand whether these results are representative for the whole setting or whether these results could be very local would be helpful for the reader to understand whether this is applicable to other systems.

Author reply: We have specified why we work from one drill-site. This is out of necessity: no other drill site recovered deeper strata and U1595 was the only site at which Unit L3 was recovered. With regards to the conceptual model, we now also specify what this means for the interpretations of the paleo-hydrothermal system.

"Unit L3 is present in seismic-reflection profiles across the entire caldera[47]." And: "A larger hydrothermal source could have been achieved either by having more widespread low-temperature, diffuse vents (as for the present day)[7,8,18,25,31] across the caldera, or by higher-temperature flow transporting more metals to the seafloor[35,36,40] (Fig. 5). Since IODP drilling only reached 55 mbsf at site U1595, we are unable to distinguish between these hypotheses."

Reviewer 3, comment 9: *Finally, the last paragraphs of the last chapter propose two models for the paleo hydrothermal setting. Firstly, the mechanisms introduced, e.g., low T diffuse vents vs high T, are introduced abruptly and the discussion in this section is very brief. I was also missing how the low vs high T venting affects the other metal contents. The conceptual model of Fig. 5 further compares the nowadays hydrothermal system with the paleo hydrothermal system, however the text somewhat lacks this discussion. As the final concluding conceptual model (Fig. 5) mainly refers to these last paragraphs of the discussion, I would suggest to revise and extend this section as well as revise the introduction accordingly*

Author reply: The conceptual model has now been better integrated throughout the manuscript. We gave an example in our reply to comment 1 by Reviewer 3. Hopefully, this concept now flows better within the manuscript and links the other sections together too. We have clarified in the discussion, and this also addresses the more speculative enrichments as mentioned in comment 6 by Reviewer 3:

"The key observation is that we infer that elevated hydrothermal fluxes of the Santorini caldera were significantly elevated from ca. 1400 BCE to 110 BCE, compared to the modern-day at the same site, suggesting a larger hydrothermal source in the past than exists today. This is also supported by the elevated levels of metal resistance genes found in microbes in Unit L3 and evidenced by the smaller peaks and enrichments in Hg and trace metals in the shallow Unit 1 of the cores (Fig. 2 and Fig. 4) compared to Unit L3. A larger hydrothermal source could have resulted either from more widespread low-temperature, diffuse vents (as for the present day)[7,8,18,25,31] across the caldera, or by higher-temperature flow transporting more metals to the seafloor[35,36,41] (Fig. 5). Since Site U1595 was the only IODP drilling site that reached 55 mbsf, we are unable to analyse other caldera sites to distinguish between these hypotheses by quantifying the extent of the hydrothermal signal."

Finally, we have expanded the discussion as suggested. For example, with the following:

"Our results indicate that flooded calderas not only preserve evidence of past hydrothermal activity but may also act as long-term sinks for hydrothermally-derived trace metals. This accumulation of metal-enriched sediments illustrates the potential to form reservoirs that could be remobilised during later epithermal or acid-sulphate processes."

Author remarks and replies to in-text comments:

All the in-text comments from Reviewers 2 and 3 have been addressed as suggested, with the exception of a small number specifically mentioned below:

- Line 150 in R3's original manuscript (line 201 in the revised manuscript) is asking about Au and its exclusion from the dataset: Au was often below the detection limit, and no stratigraphic trends can be seen. Further, its coefficient of variability was below 0.3, which we used as a threshold for inclusion in the main text. All reliable Au results were included in Supplementary Figure 1 at R3's suggestion.
- Line 156 in R3's original manuscript (line 207 in the revised manuscript) asked about the accuracy of the depths quoted for the enrichment of metals. Most samples (1cm-scoops) were taken from soft-sediment IODP core sections that can be easily and reliably positioned to sample at half-cm precision.
- Line 206 of the original manuscript both R2 and R3 ask for it to be removed, which we have now done and rewritten it with a clearer science focus. In reply to R2's comment that says: "Others have been doing this work for years, including Takai in Japan and the below at Brothers. Reysenbach et al. 2020".
  We understand the concerns regarding scientific focus and integration. We clarify that the metagenomic dataset presented here does not represent a routine survey, but a technically challenging and very difficult task. Microbial abundances in deep subsurface environments are extremely low, making high molecular weight DNA extraction for metagenomic analysis particularly demanding. Although microbial community analysis by amplicon sequencing in hydrothermal systems are indeed well established, true deep subsurface metagenomic datasets remain rare. Because of these technical difficulties, most metagenomic studies are focused on water column or on modern seafloor samples. Our dataset derives from subsurface material with very low biomass compared to modern environments, we believe it could be the first deep subsurface analysis in the Mediterranean. To follow R2's suggestions we have substantially revised the manuscript in order to better emphasize the methodological challenges and the novelty of the present study to construct a deep subsurface metagenome. Regarding Reysenbach et al. (2020), we note that their study focused on metagenomic analysis of modern communities by collecting modern samples from the water column and the seafloor. Our study differs since we examined subsurface material (55 meters below the seafloor) with a metagenomic analysis that is really challenging and not a routine task such as in the actively venting modern systems. We have clarified with the following: "Recovering high molecular weight DNA from deep subsurface sediments is a challenging task due to low biomass and DNA degradation with depth and time. Shotgun metagenomic sequencing enabled screening for genes involved in metal resistance and redox transformations."
- Line 500 in the original manuscript (587 of the revised) R3 asks for clarification on the elements chosen in the second run of ICP-MS. The main reason for including extra elements was because we wanted a measurement for Al to use it for normalisation in marine sediments, which is standard practice. The rest were to increase stratigraphic resolution and expand the dataset but they did not turn out to show much variation downcore (coefficient of variation for most of the 'new' elements was below 0.3) so they are not the main focus of the manuscript and therefore it was not necessary to repeat them in the samples we had previously run. This is mentioned in the main text: "For both ICP-MS runs, only elements that showed a coefficient of variation over 0.3 were included in graphs and comparisons."
- Line 592, we have edited the description of accuracy and certified reference materials to improve clarity. We have now clarified why there were a couple of higher errors for the certified materials (this is not the case for the samples; they had low errors), which was due to the fact that the certified materials had concentrations very close to the detection or

quantification limit for those elements. For example: “In the first ICP-MS run, all elements analysed were above their respective detection limits. Percentage error for certified reference materials (BCR-2, SLRS-6) was ≤13.7% for all elements except for As, V and Zn. As was ±17.9%, however, its certified value was below the method quantification limit so a lower accuracy was expected. V and Zn had percentage errors compared to the certified value of ±20.6% and 27.1%, respectively, for standard material SLRS-6, but percentage errors of only ±4.5% and 3.5% for BCR-2…highlighting the importance of having more than one type of standard when conducting analysis.”

We thank the editor and reviewers for their further comments, which have again improved the manuscript.

We are grateful to Reviewer 1's satisfaction with the manuscript and glad it met all their requirements. We appreciate that both Reviewer 2 and 3 agreed that the manuscript is more cohesive and better aligned.

Our revisions have focused on further better integrating the microbial work with the metal fluxes specifically, highlighting the importance of the microbial work as well as adding in some extra discussion surrounding Fig. 5.

Reviewer 2 had nine bullet points of minor comments, and we have incorporated and addressed:

1. *Henley et al. (2024—JVGR) is a good reference with regards discolouration of seawater surrounding a submarine volcanic edifice as a precursor to eruption*. - Done

2. *Explain the dashed lines in Fig 2a in the figure caption (presumably no data?).* - Done

3. *Lines 201-203. You can get laterally 'uniform' enrichments in the metals in the caldera floor due to metal deposition via plumes in the water column. Given a stated heterogeneous distribution of metals over the caldera floor, perhaps that reflects separate subseafloor discharge areas?* – Done, clarified.

4. *Line 256. "polymetallic hydrothermal activity" is an awkward phrase. You mean 'hydrothermal activity associated with polymetailic mineralization'.* – Done, phrase revised.

5. *Are the authors aware of the microbial work done by Reysenbach et al. (2020—PNAS) associated with seafloor hydrothermal vents at Brothers volcano? Might provide further insight and/or comparison.* – Yes, we addressed this in the previous reply to reviewers.

6. *Line 280. "...pulsing of hydrothermal activity..." is described in some detail by de Ronde et al. (2005; 2011) for Brothers volcano of the Kermadec arc over a range of time scales from data related to short term vent fluid chemistry, sulphide isotopes, etc. Similalry, Stucker et al. (2023—Econ. Geol.) for vent fluids over a greater time interval, and Walker & de Ronde (2023—Econ. Geol.) for hydrothermal plumes over the volcano. All point to 'pulsed' contributions to the hydrothermal system, or at least episodes of more (or less) vigouous activity, especially w.r.t. magmatic contributions. Is this the link between the work done by the authors of metal fluxes and the microbes??* – Done, these references are included and referred to. The link between microbes and metal fluxes is addressed in more detail in the replies below.

7. *Line 325. I would agree with this statement only it appears to contradict a previous statement in line 202?* – Done, revised.

8. *Line 335. This sentences corroborates the work done by de Ronde et al. (2005; 2011) and others have said same to have occurred at Brothers volcano.* – Done, referenced.

9. *Line 343. You have a 'z' in the formula but refer to 'h' in the text for depth down the core. Be consistent.* – Done, revised.

Please see below a point-by-point reply of the suggested revisions (in numerical order R2-R3, R1 had no comments):

<u>Reviewer 2</u>

<u>Reviewer 2 comment 1:</u> *However, my overriding comment is similar to what I said in my previous review. That is, the connection between the hydrothermal results and metal fluxes, and the microbial work, appears disjointed and not that strong?...What is the main relevance of the microbial population described? How is this important?*

<u>Author reply:</u> The microbial work is to support and elevate the metal fluxes and enrichments. No system operates in isolation, and we thought it was important to show the tandem responses of both the organic and inorganic components of the hydrothermal system in Santorini. That said, we have added in some text to make this relationship and the reason for the co-inclusion in the manuscript clearer. In the final version of the manuscript, we have clarified that the metagenomic analysis provides a bio-signature of sustained hydrothermal activity, recording the effect of metal-rich fluxes and providing evidence for what type of hydrothermal activity the pre-726 CE hydrothermal system might have exhibited. This could not have been elucidated from the metal fluxes alone. We have added into the introduction:

"Microorganisms can act as sensitive recorders of environmental conditions, and may directly influence the fate of hydrothermally derived elements[2]. Thus, understanding how microbial communities respond to hydrothermal perturbations improves our ability to interpret marine, seafloor and subsurface biogeochemical processes."

And into the discussion:

"However, since we see gene abundances for the Mn transport and Co/Zn/Cd efflux system surpassing the levels found in both present-day Santorini and at Kolumbo, which is a vigorous, high-temperature system, it is likely that the pre-726 CE hydrothermal system in Santorini was driven by focused, high-temperature activity, similar to that of present-day Kolumbo."

And:

"The parallel enrichment of hydrothermal metals and microbial adaptations demonstrate that hydrothermal activity was sufficiently intense and persistent."

<u>Reviewer 3</u>

<u>Reviewer 3 comment 1</u>: *As a minor remaining comment, I still think that the discussion of the two possible hydrothermal models (more widespread low-temperature diffuse venting vs. higher-temperature focused venting) remains somewhat vague. At the moment, the concept is being introduced and discussed very briefly. The authors state that this cannot currently be resolved without additional data, which is reasonable. However it would help the reader if the authors could elaborate slightly further on what processes or controls may favors these two styles of venting and whether the current observations lean more towards one interpretation (for example based on implications from other hydrothermal systems). In this context, the conceptual model shown in Fig. 5 could also be integrated a bit more strongly into the main text. That said, I understand that this is not the primary focus of the manuscript.*

Author reply: We have now provided context on what processes would control each outcome of the conceptual model and we have linked the conceptual model to a more data-based interpretation with the integration of the microbiology as evidence for a potentially higher-temperature, Kolumbo-like activity during the pre-726 CE hydrothermal system, providing more of a ‘conclusion’ as Reviewer 3 alluded to. In the discussion of the conceptual model, we have added:

“Whether a hydrothermal system exhibits venting more characterised by one end member or the other will be controlled by a variety of factors such as permeability, seawater entrainment and magma supply [16].

And:

“However, since we see gene abundances for the Mn transport and Co/Zn/Cd efflux system surpassing the levels found in both present-day Santorini and at Kolumbo, which is a vigorous, high-temperature system, it is likely that the pre-726 CE hydrothermal system in Santorini was driven by focused, high-temperature activity, similar to that of present-day Kolumbo.”

Review Nature Communications

The authors used drilled sediments from the Santorini Caldera to reconstruct the timescales of hydrothermal activity and combined these findings with trace element analysis and biological markers to estimate metal budgets/fluxes. I agree with the methods applied in the manuscript and the main conclusions revealing multiple pulses of hydrothermal activity throughout ~1100 yrs. The manuscript is mostly well written and conclusive, however I propose some essential revisions below. In addition to the more essential revisions that I have suggested below, I have added minor comments to the attached manuscript.

Generally, I think the different sections of the manuscript could be better related and aligned, for example the abstract, introduction and conclusion. From the abstract, I expected a bigger focus on the dynamics of the paleo-hydrothermal system and how it relates to metal enrichments. The introduction then brings up different tectonic settings where hydrothermal systems occurs, however they are not further discussed in regard to the results of the manuscript. The metal resources are barely mentioned there. The conclusion and the final model (Fig. 5) then suddenly focuses on different possible conceptual models of the paleo hydrothermal system, which were neither mentioned in the abstract nor in the introduction. Additionally, the section regarding the microbiology is not very well connected to the geology. I understand that an expedition includes a lot of different working groups, however at the current state, this section is not really contributing to the main conclusions of the manuscript except for the hydrothermal activity marker. It is fine to include it, however I think it should be better integrated and combined with the geology if possible.

I would therefore strongly suggest to revise the different sections of the manuscript accordingly, so that the sections are better connected and correlate in regard to the main manuscript messages.

The introduction in general lacks some information relevant to the manuscript. For example, the final conceptual model compares the active system with the paleosystem, so the introduction could include the current hydrothermal activity at Santorini, what are the temperature ranges, what are the metal contents and estimations, precipitation conditions, what minerals (ores) occur ect? This is also interesting in respect to the metal budget and modelling of fluxes. One of the final conclusions refers to low- vs high temperature venting and therefore should be explained in the introduction. I have also added some comments to the manuscript.

Regarding the sampling and methods. It seems to me that the lower parts of the drill cores, so L4 and L5, are underrepresented in the sampling. I understand that the recovery appears to be worse, however based on the recovery map (in black), there are quite some recoveries also in the deeper parts. However for the trace element analysis, not all of them were selected and I wonder why. This results in quite a big sampling gap at depth. This should be explained somewhere. Vice versa, why was the depth of 55-60 mbsf so strongly sampled?

Also as this is a hydrothermal vent field, the manuscript would benefit from something in the method about which part of the sediments were used for the trace element analysis. The method section states how much g per sample was used but not how one sample is defined. Maybe I am wrong here, but I would guess that the deviation of metals is quite inhomogeneous throughout the sediment. So how did you handle this and how does this affect the metal budgeting? Moreover, the manuscript focuses on the metal budget itself, but it is for example missing how these metals are deposited in the sediments. For further minor comments regarding the method, please see the comments in the manuscript.

Regarding the evolution of the hydrothermal system I have a few remarks and have also included some in the manuscript. I had trouble to understand how the three phases of hydrothermal activity were defined. There are more than the 6 peaks throughout the depth, there are not three known eruptions but two, and they do not correlate with the cold/wet periods. Was Hg used as a threshold? It would really help the reader to better understand how these phases were defined, so I would suggest to include this in the manuscript. I was also missing a discussion on the shallower peaks? I think the MS would additionally benefit from a comparison to some literature data here to understand what the sediment signature is, the ocean floor signature and the hydrothermal enrichment. The introduction mentions some literature values of Hg in other hydrothermal systems and it would be nice to have this comparison here. I have also suggested in the MS to add some of these references to the Fig. 4.

Generally, one large aspect of the manuscript that I am missing is the integration of the results into the tectonic setting and how these results compare to other hydrothermal systems. The manuscript suggests large implications for hydrothermal systems and metal enrichments, however without context to other systems, it rather reads like a case study. I agree about the importance of understanding the timeframes of hydrothermal systems, which is why I would suggest to extend the discussion in this respect. The integration of some literature data would increase the significance of this manuscript by giving the reader some context to other hydrothermal systems. I would suggest to include the tectonic setting into the discussion. The introduction mentions the better-studied ocean ridge vents compared to the understudied subduction zone vents and I was then wondering throughout reading the different chapters, what differences may be expected and how this related to the results. Especially regarding the evolution of the paleo-hydrothermal system and the suggested prolonged paleo-hydrothermal system, it would be very interesting to understand whether the tectonic setting has an influence and whether we can expect similar results e.g., for ocean ridge vent fields (but also for other hydrothermal systems in Greece). I think this would contribute to the applicability to more studies of hydrothermal systems and hence increase the significance of the manuscript. Additionally, the manuscript would benefit from including literature data in most of the discussion sections. I have already mentioned above, that e.g., a comparison to literature values of Hg would be good. I would suggest the same for the metal concentrations. Here, the manuscript only mentions a 4-time enrichment and it would be beneficial to give some more details about the enrichment, for example what the known enrichments are in other hydrothermal systems nowadays and paleosystems, what about other systems in Greece. There is one comparison to Tonga and the authors state that Tonga has a 5 times higher Hg flux, but they offer no explanation why this might occur. Does this mean the system in Tonga was active even longer? Again, how the results from Santorini are applicable to other hydrothermal systems would increase the studies significance. Lastly, this manuscript is mainly working with samples from one drill site and I would suggest to add a remark –whether in the manuscript or the supplement- about the variations across the caldera. To understand whether these results are representative for the whole setting or whether these results could be very local would be helpful for the reader to understand whether this is applicable to other systems.

Finally, the last paragraphs of the last chapter propose two models for the paleo hydrothermal setting. Firstly, the mechanisms introduced, e.g., low T diffuse vents vs high T, are introduced abruptly and the discussion in this section is very brief. I was also missing how the low vs high T venting affects the other metal contents. The conceptual model of Fig. 5 further compares the nowadays hydrothermal system with the paleo hydrothermal system, however the text somewhat lacks this discussion. As the final concluding conceptual model (Fig. 5) mainly refers to these last paragraphs of the discussion, I would suggest to revise and extend this section as well as revise the introduction accordingly
